# Structural Basis for Designing Multiepitope Vaccines Against COVID-19 Infection: In Silico Vaccine Design and Validation

**DOI:** 10.2196/19371

**Published:** 2020-06-19

**Authors:** Sukrit Srivastava, Sonia Verma, Mohit Kamthania, Rupinder Kaur, Ruchi Kiran Badyal, Ajay Kumar Saxena, Ho-Joon Shin, Michael Kolbe, Kailash C Pandey

**Affiliations:** 1 Infection Biology Group Department of Biotechnology Mangalayatan University Aligarh India; 2 Molecular Medicine Laboratory School of Life Science Jawaharlal Nehru University New Delhi India; 3 Parasite-Host Biology Group Protein Biochemistry and Engineering Lab ICMR-National Institute of Malaria Research New Delhi India; 4 Department of Biotechnology Institute of Applied Medicines and Research Ghaziabad India; 5 Department of Chemistry Guru Nanak Dev University Amritsar India; 6 Department of Economics Mangalayata University Aligarh India; 7 Department of Microbiology School of Medicine Ajou University Suwon, Gyeonggi-do Republic of Korea; 8 Centre for Structural Systems Biology Department for Structural Infection Biology Helmholtz-Centre for Infection Research Hamburg Germany; 9 Faculty of Mathematics, Informatics and Natural Sciences University of Hamburg Hamburg Germany

**Keywords:** COVID-19, severe acute respiratory syndrome coronavirus 2 (SARS-CoV-2), coronavirus, human transporter associated with antigen processing (TAP), toll-like receptor (TLR), epitope, immunoinformatics, molecular docking, molecular dynamics simulation, multiepitope vaccine

## Abstract

**Background:**

The novel coronavirus disease (COVID-19), which is caused by severe acute respiratory syndrome coronavirus 2 (SARS-CoV-2), has led to the ongoing 2019-2020 pandemic. SARS-CoV-2 is a positive-sense single-stranded RNA coronavirus. Effective countermeasures against SARS-CoV-2 infection require the design and development of specific and effective vaccine candidates.

**Objective:**

To address the urgent need for a SARS-CoV-2 vaccine, in the present study, we designed and validated one cytotoxic T lymphocyte (CTL) and one helper T lymphocyte (HTL) multi-epitope vaccine (MEV) against SARS-CoV-2 using various in silico methods.

**Methods:**

Both designed MEVs are composed of CTL and HTL epitopes screened from 11 Open Reading Frame (ORF), structural and nonstructural proteins of the SARS-CoV-2 proteome. Both MEVs also carry potential B-cell linear and discontinuous epitopes as well as interferon gamma–inducing epitopes. To enhance the immune response of our vaccine design, truncated (residues 10-153) *Onchocerca volvulus* activation-associated secreted protein-1 was used as an adjuvant at the N termini of both MEVs. The tertiary models for both the designed MEVs were generated, refined, and further analyzed for stable molecular interaction with toll-like receptor 3. Codon-biased complementary DNA (cDNA) was generated for both MEVs and analyzed in silico for high level expression in a mammalian (human) host cell line.

**Results:**

In the present study, we screened and shortlisted 38 CTL, 33 HTL, and 12 B cell epitopes from the 11 ORF protein sequences of the SARS-CoV-2 proteome. Moreover, the molecular interactions of the screened epitopes with their respective human leukocyte antigen allele binders and the transporter associated with antigen processing (TAP) complex were positively validated. The shortlisted screened epitopes were utilized to design two novel MEVs against SARS-CoV-2. Further molecular models of both MEVs were prepared, and their stable molecular interactions with toll-like receptor 3 were positively validated. The codon-optimized cDNAs of both MEVs were also positively analyzed for high levels of overexpression in a human cell line.

**Conclusions:**

The present study is highly significant in terms of the molecular design of prospective CTL and HTL vaccines against SARS-CoV-2 infection with potential to elicit cellular and humoral immune responses. The epitopes of the designed MEVs are predicted to cover the large human population worldwide (96.10%). Hence, both designed MEVs could be tried in vivo as potential vaccine candidates against SARS-CoV-2.

## Introduction

The novel coronavirus disease (COVID-19), which is caused by severe acute respiratory syndrome coronavirus 2 (SARS-CoV-2), has resulted in the ongoing outbreak of a severe form of respiratory disease leading to death with a mortality rate of 3.4% [1]. SARS-CoV-2 is a novel coronavirus associated with a respiratory disease that initiated in the city of Wuhan in Hubei province, China. The disease is highly contagious; as of March 21, 2020, it had spread to 182 countries and territories since its outbreak in China in December 2019. Worldwide, as of March 21, 2020, the total number of confirmed cases was reported to be 266,073, and the total death count was reported to be 11,184 [2]. Overall, SARS-CoV-2 infection has created a global emergency. The economic impact of COVID-19 is even harsher and has placed the world at economic risk. As of March 9, 2020, the worst case scenario was a US $2 trillion shortfall in global income, with a $220 billion impact on developing countries. The COVID-19 shock will cause a recession in several countries and depress global annual growth this year to below 2.5%, which is the recessionary threshold for the world economy [3].

The infection mechanism and pathogenesis of SARS-CoV-2 are currently largely unknown. According to the National Center for Biology Information (NCBI) protein sequence database [4], the proteome of SARS-CoV-2 is composed of 11 Open Reading Frame (ORF), structural and non-structural proteins. These include a polyprotein (ORF1ab), surface protein (S protein), ORF3, envelope protein (E protein), membrane protein (M protein), ORF6, ORF7a, ORF7b, ORF8, nucleocapsid protein (N protein), and ORF10. The actual functions and pathogenic or proliferative roles of these SARS-CoV-2 coronavirus proteins are currently largely unknown.

The SARS-CoV-2 polyprotein (ORF1ab), with a length of 7096 amino acids (AAs), is composed of 16 different expressed proteins, namely leader protein (nsp1, location: 1-180 AA); nsp2 (location: 181-818 AA); nsp3 (former nsp1, carries conserved domains: N-terminal acidic, predicted phosphoesterase, papain-like proteinase, Y-domain, transmembrane domain 1 and adenosine diphosphate-ribose 1''-phosphatase, location: 819-2763 AA); nsp4 (contains transmembrane domain 2, location: 2764-3263 AA); 3C-like proteinase (nsp5, main proteinase, mediates cleavage downstream of nsp4, location: 3264-3569 AA); nsp6 (putative transmembrane domain, location: 3570-3859 AA); nsp7 (location: 3860-3942 AA); nsp8 (location: 3943-4140 AA); nsp9 (ssRNA-binding protein, location: 4141-4253 AA); nsp10 (formerly known as growth-factor-like protein, location: 4254-4392 AA); nsp11 (location: 4393-4405 AA); RNA-dependent RNA polymerase (nsp12, location: 4393-5324 AA); helicase (nsp13; zinc-binding domain, NTPase/helicase domain, RNA 5'-triphosphatase, location: 5325-5925 AA); 3'-to-5' exonuclease (nsp14, location: 5926-6452 AA); endo RNAse (nsp15, location: 6453-6798 AA); and 2'-O-ribose methyltransferase (nsp16; location: 6799-7096 AA).

The SARS-CoV-2 coronavirus S protein is a structural protein that acts as a spike protein; its location is 21563-25384 AA, and its length is 1273 AA. The ORF3a protein is located at 25393-26220 AA, and its length is 275 AA. The E protein (ORF4) is a structural protein; its location is 26245-26472 AA, and its length is 75 AA. The M protein (ORF5) is a structural protein; its location is 26523-27191 AA, and its length is 222 AA. The ORF6 protein is located at 27202-27387 AA, and its length is 61 AA. The ORF7a protein is located at 27394-27759 AA, and its length is 121 AA. The ORF7b protein is located at 27756-27887 AA, and its length is 43 AA. The SARS-CoV-2 coronavirus ORF8 protein is located at 27894-28259 AA, and its length is 121 AA. The N protein) (ORF9) is a structural protein; its location is 28274-29533 AA, and its length is 419 AA. The ORF10 protein is located at 29558-29674 AA and has a length of 38 AA [4].

Although the exact mechanisms and roles of the abovementioned proteins of the SARS-CoV-2 coronavirus proteome are not well known, these proteins are potential candidates for use in vaccines against SARS-CoV-2 coronavirus infection. In this study, we screened high-potential epitopes from all the abovementioned proteins; further, we designed and proposed cytotoxic T lymphocyte (CTL) and helper T lymphocyte (HTL) multiepitope-based vaccine candidates against SARS-CoV-2 coronavirus infection.

## Methods

### Background

In this study on SARS-CoV-2 coronavirus, we screened potential epitopes and designed and proposed two multiepitope vaccines (MEVs) composed of screened CTL and HTL epitopes with overlapping regions of B cell epitopes. Hence, the proposed MEVs have the potential to elicit both humoral and cellular immune response. To enhance immune response, truncated (residues 10-153) *Onchocerca volvulus* activation-associated secreted protein-1 (Ov-ASP-1) was utilized as an adjuvant at the N-termini of both MEVs. The truncated Ov-ASP-1 was chosen due to its potential to activate antigen-processing cells (APCs) [5-7]. All the SARS-CoV-2 proteins mentioned in the introduction were utilized to screen potential CTL, HTL, and B cell epitopes. The screened epitopes were further studied to identify overlapping consensus regions among them. The epitopes showing regions of partial or complete overlap were chosen for further detailed studies.

The chosen CTL and HTL epitopes were analyzed for their molecular interactions with their respective human leukocyte antigen (HLA) allele binders. Moreover, the molecular interactions of the chosen CTL epitopes were analyzed for with the transporter associated with antigen processing (TAP) cavity to observe their smooth passage from the cytoplasm to the endoplasmic reticulum (ER) lumen [8,9]. Tertiary models of both MEVs were generated and refined. Both MEV models were further utilized to screen B cell linear and discontinuous epitopes as well as interferon gamma (IFNγ)-inducing epitopes.

Molecular signaling by multiple toll-like receptors is an essential component of the innate immune response against SARS-CoV-2. Because Ov-ASP-1 primarily binds APCs among human peripheral blood mononuclear cells and triggers proinflammatory cytokine production via toll-like receptor 3 (TLR3), the molecular interactions of both the CTL and HTL MEV models with TLR3 were further analyzed by molecular docking studies [10-13]. Furthermore, the codon-optimized cDNAs of both MEVs were analyzed and were found to have high levels of expression in a mammalian (human) cell line, which would facilitate in vivo expression, experimentation, and trials (see Supplementary Figure S1 in Multimedia Appendix 1).

### Screening of Potential Epitopes

#### T cell Epitope Prediction

##### Screening of CTL Epitopes

The CTL epitopes were screened using the Immune Epitope Database (IEDB) tools MHC (major histocompatibility complex)-I Binding Predictions and MHC-I Processing Predictions [14-16]. These two tools use six different methods (consensus, NN-align, SMM-align, combinatorial library, Sturniolo, and NetMHCIIpan), and they generate a percentile rank and a total score, respectively.

The screening is based on the total number of cleavage sites in the protein. The TAP score estimates an effective –log value of the half maximal inhibitory concentration (IC_50_) for binding to the TAP of a peptide or its N-terminal prolonged precursors. The MHC binding prediction score is the –log(IC_50_) value for binding to the MHC of a peptide [17]. The IC_50_ values (nanomolar) for each epitope and MHC allele binding pair were also obtained using the MHC-I Binding Predictions IEDB tool. Epitopes with high, intermediate, and low affinities of binding to their HLA allele binders have IC_50_ values of <50 nM, <500 nM, and <5000 nM, respectively.

The immunogenicities of all the screened CTL epitopes were also obtained using the MHC I Immunogenicity IEDB tool [17] with all parameters set to the default to analyze the first, second, and C-terminus amino acids of each screened epitope. The tool predicts the immunogenicity of a given peptide-MHC complex based on the physiochemical properties of its constituting amino acids and their positions within the peptide sequence.

##### Screening of HTL Epitopes

To screen out the HTL epitopes from the SARS-CoV-2 proteins, the IEDB tool MHC-II Binding Predictions was used. This tool generates a percentile rank for each potential peptide. The lower the percentile rank, the higher the affinity. This percentile rank is generated by the combination of three different methods, namely combinatorial library, SMM_align, and Sturniolo, and by comparing the score of the peptide against the scores of five million other random 15-mer peptides in the SWISS-PROT database [18-21]. The rank from the consensus of all three methods was generated by the median percentile rank of the three methods.

##### Population Coverage by CTL and HTL Epitopes

The IEDB Population Coverage tool was used to elucidate the world human population coverage by the shortlisted 38 CTL and 33 HTL epitopes derived from 9 SARS-CoV-2 proteins [22]. T cells recognize the complex between a specific major MHC molecule and a particular pathogen-derived epitope. The given epitope will only elicit a response in an individual who expresses an MHC molecule that is capable of binding that particular epitope. This denominated MHC restriction of T cell responses and the MHC polymorphism provides the basis for population coverage study. The MHC types are expressed at dramatically different frequencies in different ethnicities. Hence, a vaccine with larger population coverage could be of greater importance [21]. Clinical administration of multiple epitopes, including both CTL and HTL epitopes, is predicted here to have a higher probability of larger human population coverage worldwide.

### B Cell Epitope Prediction

#### Sequence-Based B Cell Epitope Prediction

The protein sequence–based Bepipred Linear Epitope Prediction method [23] was utilized to screen linear B cell epitopes from 11 different SARS-CoV-2 protein ORFs. The B Cell Epitope Prediction Tools of the IEDB server were utilized. In this screening, parameters such as the hydrophilicity, flexibility, accessibility, turns, exposed surface, polarity, and antigenic propensity of the polypeptides are correlated with their location in the protein. This enables a search for continuous epitopes predicted from a protein sequence. The prediction is based on the propensity scales for each of the 20 amino acids. For a window size n, i – (n – 1)/2 neighboring residues on each side of residue i are used to compute the score for residue i. The Bepipred Linear Epitope Prediction method used here is based on the propensity scale method as well as the physiochemical properties of the given antigenic sequence to screen potential epitopes [23].

#### Characterization of Potential Epitopes

##### Epitope Conservation Analysis

The shortlisted CTL, HTL, and B cell epitopes screened from eleven SARS-CoV-2 proteins were analyzed for the conservancy of their amino acid sequences using the IEDB Epitope Conservancy Analysis tool. The epitope conservancy is the number of protein sequences retrieved from the NCBI protein database that contains that particular epitope. The analysis was performed against the entire respective source protein sequences of SARS-CoV-2 proteins retrieved from the NCBI protein database [24].

##### Epitope Toxicity Prediction

The ToxinPred tool was used to analyze the toxicity of the shortlisted CTL, HTL, and B cell epitopes. The tool enables the identification of highly toxic or nontoxic short peptides. The toxicity check analysis was performed using the support vector machine-based ToxinPred method using a dataset of 1805 positive sequences and 3593 negative sequences from SWISS-PROT as well as an alternative dataset comprising the same 1805 positive sequences and 12,541 negative sequences from the Translated European Molecular Biology Laboratory (TrEMBL) database [25].

##### Overlapping Residue Analysis

The overlapping residue analysis for the shortlisted 38 CTL, 33 HTL, and 12 B cell linear epitopes was performed using multiple sequence alignment analysis with the European Bioinformatics Institute’s Clustal Omega tool [26]. The Clustal Omega multiple sequence alignment tool virtually aligns any number of protein sequences and delivers an accurate alignment.

##### Selection of Epitopes for Molecular Interaction Studies With HLA Alleles and the TAP Transporter

Based on the overlapping residue analysis of the shortlisted CTL, HTL, and linear B cell epitopes, a few CTL and HTL epitopes were chosen for further analysis. The chosen epitopes are circled in Supplementary Figure S10 (Multimedia Appendix 1). These epitopes were chosen based on their partial or full overlapping sequence regions among all three types of epitopes (CTL, HTL, and B cell). The chosen epitopes were further analyzed for their interactions with their respective HLA allele binders and TAP cavity interactions.

### Molecular Interaction Analysis of the Selected Epitopes with HLA Alleles and the TAP Transporter

#### Tertiary Structure Modeling of HLA Alleles and Selected T Cell Epitopes

SWISS-MODEL [27] was used for homology modeling of the HLA class I and II allele binders of the chosen epitopes. The amino acid sequences of the HLA allele binders were retrieved from the Immuno Polymorphism Database (IPD-IMGT/HLA). Templates for homology modeling were chosen based on the highest amino acid sequence similarity. All the generated HLA allele models had acceptable QMEAN values (cutoff -4.0) (Supplementary Table S1, Multimedia Appendix 1). The QMEAN value gives a composite quality estimate involving both global and local analysis of the model [28].

PEP-FOLD 2.0 [29], a de novo structure prediction tool available at RPBS Web Portal, was utilized to generate tertiary structures for the chosen CTL and HTL epitopes.

#### Molecular Interaction Analysis of Chosen CTL and HTL Epitopes With HLA Alleles

The PatchDock tool was utilized for in silico molecular docking studies of the selected CTL and HTL epitopes with their respective HLA class I and II allele binders. PatchDock utilizes an algorithm for unbound (real-life) docking of molecules for protein-protein complex formation. The algorithm carries out rigid docking, and the surface variability/flexibility is implicitly addressed through liberal intermolecular penetration. The algorithm focuses on the initial molecular surface fitting on localized, curvature-based surface patches, the use of geometric hashing and pose clustering for initial transformation detection, computation of shape complementarity utilizing Distance Transform, efficient steric clash detection and geometric fit scoring based on multiresolution shape representation, and utilization of biological information by focusing on hotspot-rich surface patches [30-32].

#### Molecular Interaction Analysis of Selected CTL Epitopes With the TAP Transporter

The TAP transporter plays an important role in the presentation of a CTL epitope. From the cytosol after proteasome processing, the fragmented peptide of the foreign protein is transported to the ER through the TAP transporter. From the ER, these short peptides reach the Golgi bodies and are then presented on the cell surface [9]. Molecular interaction studies of the chosen CTL epitopes within the TAP cavity were performed by molecular docking using the PatchDock tool. For accurate prediction, the cryo-EM structure of the TAP transporter (PDB ID: 5u1d) was used by removing the antigen from the TAP cavity of the original structure [8].

### Design, Characterization, and Molecular Interaction Analysis of MEVs With Immune Receptors

#### Design of the MEVs

The screened and shortlisted high-scoring 38 CTL and 33 HTL epitopes were utilized to design CTL and HTL MEVs (Tables 1 and 2). Two short peptides, EAAAK and GGGGS, were used as rigid and flexible linkers, respectively (Supplementary Figure S2, Multimedia Appendix 1). The GGGGS linker provides proper conformational flexibility to the tertiary structure of the vaccine and hence facilitates stable conformation of the vaccine. The EAAAK linker facilitates domain formation and hence aids the vaccine to obtain its final stable structure. Truncated Ov-ASP-1 protein was utilized as an adjuvant at the N termini of both the CTL and HTL MEVs [5-7,33-37].

#### Characterization of the Designed MEVs

##### Physicochemical Property Analysis of the Designed MEVs

The ProtParam tool [38] was utilized to analyze the physiochemical properties of the amino acid sequences of the designed CTL and HTL MEVs. The ProtParam analysis performs an empirical investigation of the amino acid sequence in a given query. ProtParam computes various physicochemical properties derived from a given protein sequence.

##### IFNγ-Inducing Epitope Prediction

From the designed amino acid sequences of both MEVs, potential IFNγ epitopes were screened by the IFN epitope server using a hybrid motif and support vector machine approach; the motif-based method used was MERCI (Motif-EmeRging and with Classes-Identification). This tool predicts peptides from protein sequences that have the capacity to induce IFNγ release from CD4^+^ T cells. This module generates overlapping peptides from the query sequence and predicts IFNγ-inducing peptides. For the screening, the IEDB database was used with 3705 IFNγ-inducing and 6728 non–IFNγ-inducing MHC class II binders [39,40].

##### MEV Allergenicity and Antigenicity Prediction

Both the designed MEVs were further analyzed for allergenicity and antigenicity prediction using the AlgPred [41] and VaxiJen [42] tools, respectively. The AlgPred prediction is based on the similarity of an already known epitope with any region of the submitted protein. To screen allergenicity, the SWISS-PROT data set consisting of 101,725 non-allergens and 323 allergens was used. VaxiJen utilizes an alignment-free approach that is based solely on the physicochemical properties of the query amino acid sequence. To predict the antigenicity, VaxiJen uses bacterial, viral, and tumor protein datasets to derive models for the prediction of the antigenicity of a whole protein. Every set consists of known 100 antigens and 100 nonantigens.

#### Tertiary Structure Modeling, Refinement, and Validation of the MEVs

The tertiary structures of the designed CTL and HTL MEVs were generated by homology modeling using the I-TASSER modeling tool [43]. I-TASSER is a protein structure prediction tool that is based on the sequence-to-structure-to-function paradigm. The tool generates 3D atomic models from multiple threading alignments and iterative structural assembly simulations for a submitted AA sequence. I-TASSER is based on the structure templates identified by LOMETS, a metaserver from the Protein Data Bank (PDB) library. I-TASSER only uses the template with the highest Z-score, which is the difference between the raw and average scores in the unit of standard deviation. For each target model, the I-TASSER simulations generate a large ensemble of structural conformations, called decoys. To select the final models, I-TASSER uses the SPICKER program to cluster all the decoys based on their pairwise structure similarity and reports up to 5 models. A normalized Z-score >1 indicates a good alignment and vice versa. The Cov represents the coverage of the threading alignment and is equal to the number of aligned residues divided by the length of the query protein. Ranking of template proteins is based on the TM-score of the structural alignment between the query structure model and known structures. The root mean square deviation (RMSD) is the RMSD between template residues and query residues that are structurally aligned by the TM-align algorithm.

Both the generated MEV models were refined using the ModRefiner [44] and GalaxyRefine [45] tools. The TM-score generated by ModRefiner indicates the structural similarity of the refined model to the original input model. The closer the TM-score to 1, the greater the similarity of the original and refined models. The RMSD of the refined model shows the conformational deviation from the initial input models.

The GalaxyRefine tool refines the query tertiary structure by repeated structure perturbation and by using the subsequent structural relaxation by the molecular dynamics simulation. The GalaxyRefine tool generates reliable core structures from multiple templates and then rebuilds unreliable loops or termini using an optimization-based refinement method [46,47]. To avoid any breaks in the 3D model, GalaxyRefine uses the triaxial loop closure method. The MolProbity score generated for a given refined model indicates the log-weighted combination of the clash score, the percentage of Ramachandran unfavored residues and the percentage of bad side chain rotamers.

#### Validation of the Refined Models of the CTL and HTL MEVs

The refined CTL and HTL MEV 3D models both were further validated by the RAMPAGE analysis tool [48,49]. The generated Ramachandran plots for the MEV models show the sterically allowed and disallowed residues along with their dihedral psi (ψ) and phi (φ) angles.

#### Linear and Discontinuous B-cell Epitope Prediction of the MEVs

The ElliPro antibody epitope prediction tool available at the IEDB was used to screen the linear and discontinuous B cell epitopes from the MEV models. The ElliPro method analyses are based on the location of a residue in the 3D structure of a protein. For example, the residues lying outside an ellipsoid covering 90% of the inner core protein residues score the highest protrusion index (PI) of 0.9. The discontinuous epitopes predicted by the ElliPro tool are clustered based on the distance R in angstroms between the centers of mass of two residues lying outside the largest possible ellipsoid. A larger value of R indicates that more distant residues (residue discontinuity) are screened in the epitopes [50,51].

### Molecular Interaction Analysis of MEVs With an Immunological Receptor

#### Molecular Docking Studies of the MEVs and TLR3

Molecular interaction analysis of both designed MEVs with TLR3 was performed by molecular docking and molecular dynamics simulations. Molecular docking was performed using the PatchDock server [32]. PatchDock utilizes an algorithm for unbound docking of molecules (mimicking the real world environment) for protein-protein complex formation, as explained earlier [30,31]. For molecular docking, the 3D structure of the human TLR3 ectodomain was retrieved from the PDB (PDB ID: 2A0Z). The study provides the dynamical properties of the designed system with the MEV-TLR3 complexes and guesses at the interactions between the molecules; also, it gives exact predictions of bulk properties, including hydrogen bond formation and the conformation of the molecules forming the complex.

#### Molecular Dynamics Simulation Studies of the MEVs and the TLR3 Complex

The MEV-TLR3 molecular interactions were further evaluated using molecular dynamics simulations. The molecular dynamics simulations were performed for 10 nanoseconds using YASARA (Yet Another Scientifc Artifcial Reality Application) [52]. The simulations were performed in an explicit water environment in a dodecahedron simulation box at a constant temperature (298 kelvin) and pressure (1 atmosphere) at pH 7.4 with a periodic cell boundary condition. The solvated systems were neutralized with counterions (sodium chloride, concentration 0.9 molar). The AMBER14 force field was applied to the systems during the simulations [53,54]. Long-range electrostatic energies and forces were calculated using the particle mesh–based Ewald method [55]. The solvated structures were minimized by the steepest descent method at a temperature of 298 K and a constant pressure. Then, the complexes were equilibrated for a period of 1 nanosecond. After equilibration, a production molecular dynamics simulation was run for 10 ns at a constant temperature and pressure, and time frames were saved every 10 picoseconds for each simulation. The RMSD and root mean square fluctuation (RMSF) values for the alpha carbon (C_α_) atoms, backbone atoms, and all the atoms of both MEV complexes were analyzed for each simulation conducted.

### Generation and Analysis of cDNA of the MEVs

cDNAs of both MEVs, codon-optimized for expression in a mammalian (human) cell line, were generated using the Java Codon Adaptation Tool. The generated cDNAs of both the MEVs were further analyzed by the GenScript Rare Codon Analysis Tool. This tool analyzes the GC content, codon adaptation index (CAI) and tandem rare codon frequency for a given cDNA [56,57]. The CAI indicates the possibility of cDNA expression in a chosen expression system. The tandem rare codon frequency indicates the presence of low-frequency codons in a given cDNA.

## Results

### Screening of Potential Epitopes

#### T Cell Epitope Prediction

##### Screening of CTL Epitopes

CTL epitopes were screened using the MHC-I Binding Predictions and MHC-I Processing Predictions IEDB tools. These epitopes were shortlisted based on the total number of cleavage sites in the protein, low IC_50_ (nM) values for epitope-HLA class I allele pairs, and binding to the TAP cavity.

The 38 epitopes predicted by the MHC-I Binding Predictions tool with the highest percentile ranks were shortlisted for MEV design and are listed in Table 1. The remaining 101 epitope-HLA I allele pairs are listed in Supplementary Table S8 (Multimedia Appendix 1). The 67 epitope-HLA I allele pairs predicted by the MHC-I Processing Predictions tool with the highest total scores are listed in Supplementary Table S9 (Multimedia Appendix 1).

The immunogenicities of the shortlisted CTL epitopes were also determined and are noted in Table 1 and in Supplementary Tables S8 and S9 (Multimedia Appendix 1). A higher immunogenicity score indicates greater immunogenic potential of the given epitope.

**Table 1:** Characteristics of the shortlisted high-percentile-ranking SARS-CoV-2 CTL epitopes and their respective HLA allele binders.

SARS-CoV-2^a^ protein	Peptide length, amino acids	Peptide sequence	Conservancy (%)	Immunogenicity	Toxicity	Allele	Methods used^b^	Percentile rank
E protein^c^	9	LLFLAFVVF	480/482 (99.59)	0.2341	Nontoxic	B15:01	Consensus (ann/smm/comblib_sidney 2008)	0.1
E protein	9	LTALRLCAY	478/482 (99.17)	0.01886	Nontoxic	A01:01	Consensus (ann/smm)	0.12
M protein^d^	11	YFIASFRLFAR	474/477 (99.37)	0.19709	Nontoxic	A33:01	ann	0.03
M protein	10	ATSRTLSYYK^e^	472/477 (98.95)	–0.13563	Nontoxic	A11:01	Consensus (ann/smm)	0.06
N protein^f^	9	MEVTPSGTW	485/498 (97.39)	–0.06279	Nontoxic	B44:02	Consensus (ann/smm)	0.06
N protein	9	KPRQKRTAT	487/498 (97.79)	–0.20542	Nontoxic	B07:02	Consensus (ann/smm/comblib_sidney 2008)	0.1
orf10	9	MGYINVFAF	477/480 (99.38)	–0.09452	Nontoxic	B35:01	Consensus (ann/smm/comblib_sidney 2008)	0.1
orf10	10	GYINVFAFPF^e^	232/236 (98.31)	0.20158	Nontoxic	A23:01	Consensus (ann/smm)	0.11
orf-1ab	11	SEMVMCGGSLY	452/456 (99.12)	0.32633	Nontoxic	B44:02	ann	0.03
orf-1ab	11	FYWFFSNYLKR	455/456 (99.78)	0.37766	Nontoxic	A33:01	ann	0.04
orf-1ab	8	ISNSWLMW	454/456 (99.56)	–0.24791	Nontoxic	B58:01	ann	0.05
orf-1ab	10	ETISLAGSYK	455/456 (99.78)	0.08174	Nontoxic	A68:01	Consensus (ann/smm)	0.06
orf-1ab	9	QEILGTVSW	455/456 (99.78)	0.27341	Nontoxic	B44:02	Consensus (ann/smm)	0.06
orf-1ab	9	STFNVPMEK	456/456 (100.00)	–0.32016	Nontoxic	A11:01	Consensus (ann/smm)	0.06
orf-1ab	10	RMYIFFASFY	456/456 (100.00)	0.21107	Nontoxic	A30:02	Consensus (ann/smm)	0.06
orf-1ab	10	FLFVAAIFYL	454/456 (99.56)	–0.19814	Nontoxic	A02:01	Consensus (ann/smm)	0.06
orf-1ab	10	RYFRLTLGVY	456/456 (100.00)	0.03976	Nontoxic	A30:02	Consensus (ann/smm)	0.06
orf-1ab	9	FLNGSCGSV	456/456 (100.00)	–0.20585	Nontoxic	A02:03	Consensus (ann/smm)	0.06
orf-1ab	9	CTDDNALAY	476/479 (99.37)	0.32004	Nontoxic	A01:01	Consensus (ann/smm)	0.06
orf-1ab	10	CTDDNALAYY^e^	476/479( 99.37)	0.28694	Nontoxic	A01:01	Consensus (ann/smm)	0.06
orf-1ab	11	MYKGLPWNVVR	456/456 (100.00)	–0.11151	Nontoxic	A33:01	ann	0.06
orf-1ab	10	SIINNTVYTK^e^	456/456 (100.00)	0.15936	Nontoxic	A11:01	Consensus (ann/smm)	0.06
orf-1ab	10	LPVNVAFELW	450/456 (98.68)	–0.00254	Nontoxic	B53:01	Consensus (ann/smm)	0.06
orf-1ab	9	DEWSMATYY^e^	455/456 (99.78)	0.07355	Nontoxic	B44:03	Consensus (ann/smm)	0.07
orf-1ab	10	YILFTRFFYV	454/456 (99.56)	–0.02845	Nontoxic	A02:06	Consensus (ann/smm)	0.07
orf-1ab	10	YIFFASFYYV	456/456 (100.00)	0.12661	Nontoxic	A02:06	Consensus (ann/smm)	0.07
ORF3a	9	YLYALVYFL^e^	456/456 (100.00)	0.40924	Nontoxic	A02:01	Consensus (ann/smm/comblib_sidney 2008)	0.1
ORF3a	10	IPYNSVTSSI	454/456 (99.56)	0.13772	Nontoxic	B51:01	Consensus (ann/smm)	0.11
Orf6	8	RTFKVSIW	466/481 (96.88)	0.13151	Nontoxic	B57:01	ann	0.05
Orf6	11	AEILLIIMRTF	471/481 (97.92)	–0.32835	Nontoxic	B44:02	ann	0.06
ORF7a	8	RARSVSPK	480/481 (99.79)	–0.18221	Nontoxic	A30:01	ann	0.11
ORF7a	10	QLRARSVSPK	479/481 (99.58)	0.1815	Nontoxic	A03:01	Consensus (ann/smm)	0.16
orf7b	9	FLAFLLFLV	472/480 (98.33)	–0.16177	Nontoxic	A02:03	Consensus (ann/smm)	0.07
orf8	9	HFYSKWYIR	472/480 (98.33)	–0.27456	Nontoxic	A31:01	Consensus (ann/smm)	0.11
S protein^g^	10	WTAGAAAYYV	470/472 (99.58)	0.15455	Nontoxic	A68:02	Consensus (ann/smm)	0.06
S protein	10	FPNITNLCPF	472/472 (100.00)	0.1009	Nontoxic	B53:01	Consensus (ann/smm)	0.06
S protein	10	NYNYLYRLFR	465/472 (98.52)	0.08754	Nontoxic	A33:01	Consensus (ann/smm)	0.07
S protein	8	NYLYRLFR	465/472 (98.52)	0.13144	Nontoxic	A33:01	ann	0.07

^a^SARS-CoV-2: severe acute resipiratory syndrome coronavirus 2.

^b^Methods: ann: artificial neural network. Comblib_sidney2008: combinatorial peptide libraries [19]. smm: stabilized matrix method.

^c^E protein: envelope protein.

^d^M protein: membrane protein.

^e^Matches a recently published epitope, indicating consensus with results [58].

^f^N protein: nucleocapsid protein.

^g^S protein: surface protein.

##### Screening of HTL Epitopes

The screening of HTL epitopes from 11 different SARS-CoV-2 ORF proteins was performed based on percentile rank. The smaller the percentile rank, the higher the affinity of the peptide to its respective HLA allele binders. The 33 epitopes with high percentile ranking were shortlisted (Table 2). An additional 180 potential HTL cell epitope-HLA allele II pairs with high percentile ranks screened in our study are listed in Supplementary Table S10 (Multimedia Appendix 1).

**Table 2:** Characteristics of the shortlisted high-scoring SARS-CoV-2 HTL epitopes and their respective HLA allele binders.

SARS-CoV-2^a^ protein	Peptide	Conservancy (%)	Toxicity	Alleles	Methods used^b^	Percentile rank
E protein^c^	LLFLAFVVFLLVTLA	480/482 (99.59)	Nontoxic	DPA1-03:01/DPB1-04:02	Consensus (comb.lib./smm/nn)	0.02
E protein	VLLFLAFVVFLLVTL	480/482 (99.59)	Nontoxic	DPA1-03:01/DPB1-04:02	Consensus (comb.lib./smm/nn)	0.02
M protein^d^	GLMWLSYFIASFRLF	465/477 (97.48)	Nontoxic	DPA1-01:03/DPB1-02:01	Consensus (comb.lib./smm/nn)	0.05
M Protein	LMWLSYFIASFRLFA	466/477 (97.69)	Nontoxic	DPA1-01:03/DPB1-02:01	Consensus (comb.lib./smm/nn)	0.05
M protein	LSYYKLGASQRVAGD^e^	472/477 (98.95)	Nontoxic	DRB1-09:01	Consensus (comb.lib./smm/nn)	0.06
N protein^f^	AQFAPSASAFFGMSR	486/498 (97.59)	Nontoxic	DRB1-09:01	Consensus (comb.lib./smm/nn)	0.01
N protein	IAQFAPSASAFFGMS	485/498 (97.39)	Nontoxic	DRB1-09:01	Consensus (comb.lib./smm/nn)	0.01
N protein	PQIAQFAPSASAFFG	485/498 (97.39)	Nontoxic	DRB1-09:01	Consensus (comb.lib./smm/nn)	0.01
ORF1ab	AIILASFSASTSAFV	456/456 (100.00)	Nontoxic	DRB1-09:01	Consensus (comb.lib./smm/nn)	0.01
ORF1ab	ESPFVMMSAPPAQYE^e^	456/456 (100.00)	Nontoxic	DRB1-01:01	Consensus (comb.lib./smm/nn)	0.01
ORF1ab	IILASFSASTSAFVE	456/456 (100.00)	Nontoxic	DRB1-09:01	Consensus (comb.lib./smm/nn)	0.01
ORF1ab	QESPFVMMSAPPAQY	456/456 (100.00)	Nontoxic	DRB1-01:01	Consensus (comb.lib./smm/nn)	0.01
ORF1ab	SPFVMMSAPPAQYEL	456/456 (100.00)	Nontoxic	DRB1-01:01	Consensus (comb.lib./smm/nn)	0.01
ORF3a	FVRATATIPIQASLP	478/481 (99.37)	Nontoxic	DPA1-02:01/DPB1-14:01	NetMHCIIpan	0.12
ORF3a	LLFVTVYSHLLLVAA	467/481 (97.08)	Nontoxic	DRB1-01:01	Consensus (comb.lib./smm/nn)	0.1
ORF6	FKVSIWNLDYIINLI	478/481 (99.38)	Nontoxic	DQA1-01:01/DQB1-05:01	Consensus (comb.lib./smm/nn)	0.02
ORF6	KVSIWNLDYIINLII	478/481 (99.38)	Nontoxic	DQA1-01:01/DQB1-05:01	Consensus (comb.lib./smm/nn)	0.02
ORF6	TFKVSIWNLDYIINL^e^	478/481 (99.38)	Nontoxic	DQA1-01:01/DQB1-05:01	Consensus (comb.lib./smm/nn)	0.02
ORF7a	IILFLALITLATCEL	479/480 (99.79)	Nontoxic	DRB1-01:01	Consensus (comb.lib./smm/nn)	0.16
ORF7a	ILFLALITLATCELY	479/480 (99.79)	Nontoxic	DRB1-01:01	Consensus (comb.lib./smm/nn)	0.16
ORF7b	CFLAFLLFLVLIMLI	231/236 (97.88)	Nontoxic	DPA1-03:01/DPB1-04:02	Consensus (comb.lib./smm/nn)	0.03
ORF7b	LCFLAFLLFLVLIML	231/236 (97.88)	Nontoxic	DPA1-03:01/DPB1-04:02	Consensus (comb.lib./smm/nn)	0.02
ORF7b	YLCFLAFLLFLVLIM	231/236 (97.88)	Nontoxic	DPA1-03:01/DPB1-04:02	Consensus (comb.lib./smm/nn)	0.02
ORF8	CTQHQPYVVDDPCPI	476/480 (99.17)	Nontoxic	DRB3-01:01	Consensus (comb.lib./smm/nn)	0.08
ORF8	HQPYVVDDPCPIHFY	476/480 (99.17)	Nontoxic	DRB3-01:01	Consensus (comb.lib./smm/nn)	0.08
ORF8	QPYVVDDPCPIHFYS	476/480 (99.17)	Nontoxic	DRB3-01:01	Consensus (comb.lib./smm/nn)	0.07
ORF10	INVFAFPFTIYSLLL	476/480 (99.17)	Nontoxic	HLA-DPA1-01:03/DPB1-02:01	Consensus (comb.lib./smm/nn)	0.29
ORF10	YINVFAFPFTIYSLL	476/479 (99.37)	Nontoxic	DPA1-01:03/DPB1-02:01	Consensus (comb.lib./smm/nn)	0.29
S protein^g^	KTQSLLIVNNATNVV	472/472 (100.00)	Nontoxic	DRB1-13:02	Consensus (smm/nn/sturniolo)	0.01
S protein	LLIVNNATNVVIKVC	469/472 (99.36)	Nontoxic	DRB1-13:02	Consensus (smm/nn/sturniolo)	0.01
S protein	QSLLIVNNATNVVIK	471/472 (99.79)	Nontoxic	DRB1-13:02	Consensus (smm/nn/sturniolo)	0.01
S protein	SLLIVNNATNVVIKV^e^	471/472 (99.79)	Nontoxic	DRB1-13:02	Consensus (smm/nn/sturniolo)	0.01
S protein	TQSLLIVNNATNVVI	471/472 (99.79)	Nontoxic	DRB1-13:02	Consensus (smm/nn/sturniolo)	0.01

^a^SARS-CoV-2: severe acute resipiratory syndrome coronavirus 2.

^b^Methods: comb.lib.: combinatorial library. nn: neural network. smm: stabilized matrix method.

^c^E protein: envelope protein.

^d^M protein: membrane protein.

^e^Matches a recently published epitope, indicating consensus with results [58].

^f^N protein: nucleocapsid protein

^g^S protein: surface protein.

#### Population Coverage by CTL and HTL Epitopes

The population coverage by the shortlisted epitopes was also studied, particularly in China, France, Italy, the United States, South Asia, East Asia, Northeast Asia, and the Middle East. From this study, we can conclude that the combined use of all the shortlisted CTL and HTL epitopes would have an average worldwide population coverage as high as 96.10% (SD 23.74) (Supplementary Table S12, Multimedia Appendix 1).

### B Cell Epitope Prediction

#### Sequence-Based B Cell Epitope Prediction

To screen B cell epitopes, we used the Bepipred Linear Epitope Prediction method. In our study, we screened 12 B cell epitopes from 11 SARS-CoV-2 ORF proteins which show partial or complete overlap with the shortlisted CTL and HTL epitopes (Table 3). An additional 206 B cell epitopes with epitope lengths of at least four AAs and a maximum of 20 AAs were screened and are listed in Supplementary Table S11, Multimedia Appendix 1.

**Table 3:** Characteristics of the shortlisted SARS-CoV-2 linear B cell epitopes obtained by the BepiPred method.

SARS-CoV-2^a^ protein	Peptide length, amino acids	Conservancy (%)	Overlapping B cell epitope	Toxicity
M protein^b^	12	471/477 (98.74)	KLGASQRVAGDS	Nontoxic
N protein^c^	42	483/498 (96.99)	RLNQLESKMSGKGQQQQGQTVTKKSAAEASK KPRQKRTATKA	Nontoxic
ORF1ab	20	455/456 (99.78)	GTTQTACTDDNALAYYNTTK	Nontoxic
ORF3a	12	478/481 (99.37)	QGEIKDATPSDF	Nontoxic
ORF3a	6	471/481 (97.92)	PYNSVT	Nontoxic
ORF7a	9	479/480 (99.79)	LYHYQECVR	Nontoxic
ORF7a	26	470/480 (97.92)	VKHVYQLRARSVSPKLFIRQEEVQEL	Nontoxic
ORF8	23	460/480 (95.83)	QSCTQHQPYVVDDPCPIHFYSKW	Nontoxic
ORF8	9	476/480 (99.17)	RVGARKSAP	Nontoxic
S protein^d^	11	470/472 (99.58)	TPGDSSSGWTA	Nontoxic
S protein	35	470/472 (99.58)	FPNITNLCPFGEVFNATRFASVYAWNRKRISNCVA	Nontoxic
S protein	62	454/472 (96.19)	NLDSKVGGNYNYLYRLFRKSNLKPFERDISTEIY QAGSTPCNGVEGFNCYFPLQSYGFQPTN	Nontoxic

^a^SARS-CoV-2: severe acute resipiratory syndrome coronavirus 2.

^b^M protein: membrane protein.

^c^N protein: nucleocapsid protein

^d^S protein: surface protein.

#### Characterization of Potential Epitopes

##### Epitope Conservation Analysis

Sequence conservation analysis of the screened CTL, HTL, and B cell epitopes showed the highly conserved nature of the shortlisted epitopes. The amino acid sequences of both the CTL epitopes and the HTL epitopes were found to be significantly conserved among the NCBI-retrieved protein sequences of SARS-CoV-2 (the CTL epitopes were 96.88%-100% conserved and the HTL epitopes were 97.08%-100% conserved; see Tables 1, 2, and 4 and Supplementary Tables S8, S9, S10, and S11, Multimedia Appendix 1).

##### Epitope Toxicity Prediction

Toxicity analyses of all the screened CTL, HTL, and B cell epitopes were also performed. The ToxinPred study of all the shortlisted epitopes showed that they all are nontoxic (Tables 1, 2, and 4; Supplementary Tables S8, S9, S10, and S11, Multimedia Appendix 1).

##### Overlapping Residue Analysis

The AA sequence overlap among the shortlisted CTL, HTL, and B cell epitopes from 11 SARS-CoV-2 ORF proteins was analyzed using the Clustal Omega multiple sequence alignment analysis tool. The analysis showed that several CTL, HTL, and B cell epitopes had overlapping AA sequences. The CTL, HTL, and B cell epitopes with two or more overlapping AA residues are shown in Supplementary Figure S3 (Multimedia Appendix 1).

##### Selection of Epitopes for Molecular Interaction Studies with HLA Alleles and the TAP Transporter

The epitopes showing overlap among the CTL, HTL, and B cell epitopes are circled in Supplementary Figure S10 (Multimedia Appendix 1) and were chosen for further study of their interactions with HLA alleles and the TAP transporter.

### Molecular Interaction Analysis of Selected Epitopes With HLA Alleles and the TAP Transporter

#### Molecular Interaction Analysis of the Chosen CTL and HTL Epitopes With HLA Alleles

**Figure 1:**
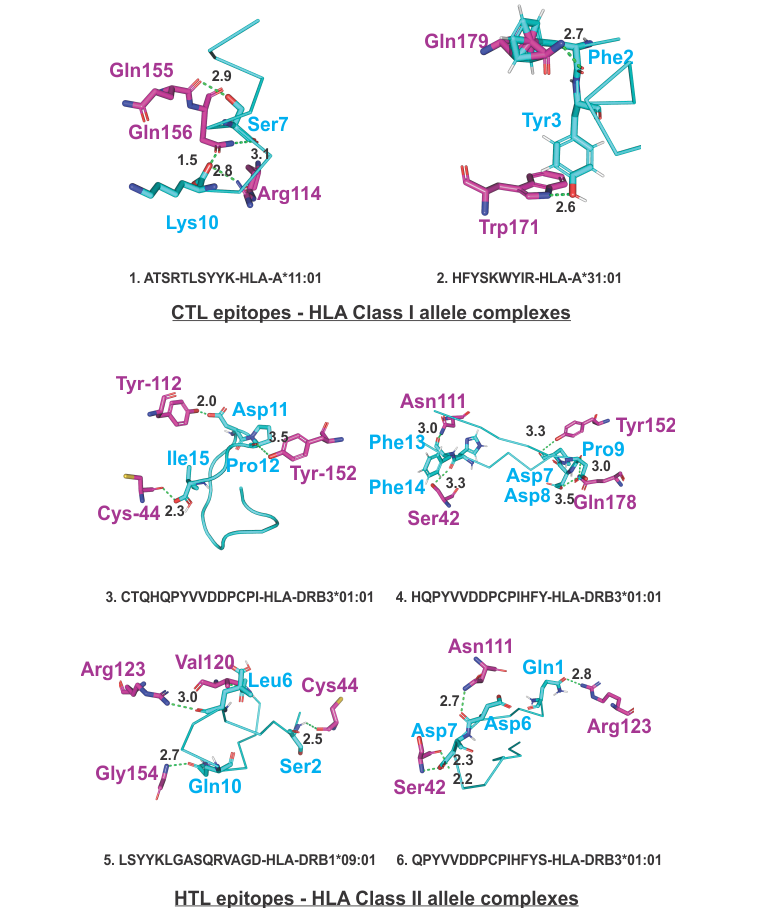
Molecular docking analysis of SARS-CoV-2 CTL epitopes and HLA alleles. Molecular docking of the chosen CTL and HTL epitopes (cyan sticks) binding the amino acid residues of their respective HLA class I and class II allele binders (magenta sticks). The study shows that the docked complexes are stable, with the formation of multiple hydrogen bonds (green dots, lengths in angstroms). CTL: cytotoxic T lymphocyte. HLA: human leukocyte antigen.

Molecular docking studies of the chosen CTL and HTL epitopes with their respective HLA class I and II allele binders were performed using the PatchDock tool. Images were generated by PyMOL [59]. The study revealed significant molecular interactions between all the chosen epitopes and their HLA allele binders, showing the formation of multiple hydrogen bonds (Figure 1). Furthermore, B-factor analysis of all the epitope–HLA allele complexes showed that the epitope ligand had a stable (blue) binding conformation in complex with the HLA allele molecule (Supplementary Figure S4, Multimedia Appendix 1). The violet-indigo-blue-green-yellow-orange-red (VIBGYOR) color presentation was used, where blue is very stable.

#### Molecular Interaction Analysis of Selected CTL Epitopes With the TAP Cavity

**Figure 2:**
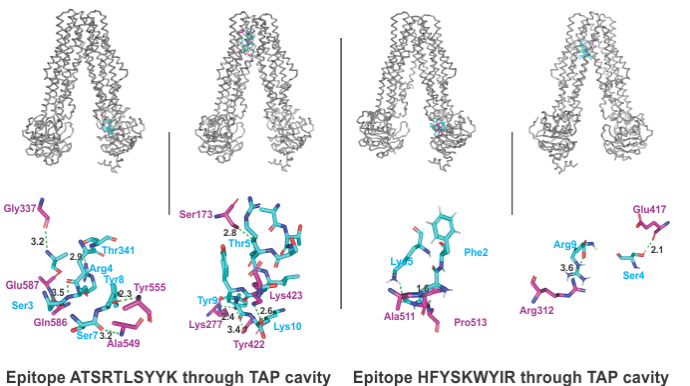
Molecular docking analysis of two CTL epitopes within the TAP transporter cavity. The molecular interactions of the CTL epitopes (cyan sticks) within the TAP cavity (gray ribbons/sticks) are shown. Detailed interactions between the residues of the epitopes and the TAP transporter residues are shown, with hydrogen bond formation indicated with green dots. H bonds are shown in green dots with lengths in angstroms. TAP: transporter associated with antigen processing.

The molecular docking interaction analysis of the chosen CTL epitopes with the TAP cavity showed significantly strong molecular interactions with the formation of several hydrogen bonds at different sites of the TAP cavity. Two sites of interaction were of particular interest: one closer to the cytoplasmic end and another closer to the ER lumen (Figure 2). This study confirms the feasibility of transportation of the chosen CTL epitopes from the cytoplasm to the ER lumen, which is an essential event for the representation of an epitope by HLA allele molecules on the surface of antigen-presenting cells.

### Characterization and Molecular Interaction Analysis of the Designed MEVs with Immune Receptors

#### Characterization of the Designed MEVs

##### Physicochemical Property Analysis of the Designed MEVs

ProtParam analysis of both the CTL and HTL MEVs was performed to analyze their physiochemical properties. The empirical physiochemical properties of the CTL and HTL MEVs are given in Table 4. The aliphatic indices and grand averages of hydropathicity of both MEVs indicate their globular and hydrophilic natures. The instability index scores of both MEVs indicates the stable nature of the protein molecules.

**Table 4:** Physicochemical property analysis based on the amino acid sequences of the designed CTL and HTL MEVs.

Property		Cytotoxic T lymphocyte multiepitope vaccine	Helper T lymphocyte multiepitope vaccine
Length (amino acids)		704	810
Molecular weight (kilodaltons)		72.62	82.80
Theoretical protrusion index		9.70	8.64
**Expected half-life (hours)**			
	*Escherichia coli*	10	10
	Yeast	30	30
	Mammalian cell	20	20
Aliphatic index		61.09	96.43
Grand average of hydropathicity		–0.090	0.501
Instability index		44.31	40.28

##### IFNγ-Inducing Epitope Prediction

IFNγ-inducing epitopes are involved in both the adaptive and the innate immune response. IFNγ-inducing 15mer peptide epitopes were screened from the amino acid sequences of the CTL and HTL MEVs using the IFNepitope server. A total of 20 CTL MEV and 20 HTL MEV INFγ-inducing positive epitopes with a score ≥1 were shortlisted (Supplementary Table S2, Multimedia Appendix 1).

##### Allergenicity and Antigenicity Prediction of the MEVs

Both the CTL and HTL MEVs were found to be nonallergenic by the AlgPred analysis (scores of –0.95185601 and –1.1293352, respectively; the threshold was –0.4). The CTL and HTL MEVs were also indicated by VaxiJen analysis to be probable antigens (prediction scores of 0.4485 and 0.4215, respectively; the default threshold is 0.4). Hence, with the mentioned analysis tools, both the CTL and HTL MEVs are predicted to be nonallergic and antigenic in nature.

#### Tertiary Structure Modeling, Refinement, and Validation of the MEVs

**Figure 3:**
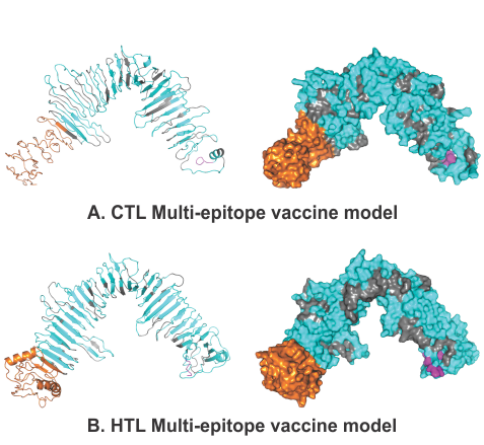
Tertiary structure modelling of the CTL and HTL multiepitope vaccines. The epitopes are shown in cyan. The adjuvant (Ov-ASP-1) is shown in orange. The linkers are shown in gray, and the 6xHis tag is shown in magenta. Cartoon and surface presentations of both the MEVs are shown. CTL: cytotoxic T lymphocyte. HTL: helper T lymphocyte.

3D homology models were generated for both the CTL and HTL MEvs using the I-TASSER modeling tool (Figure 3). The models were generated for the CTL MEV (PDB ID: 5n8pA, normal Z-score of 1.49, Cov of 0.92, TM-score of 0.916, and RMSD of 1.04 Å) and the HTL MEC (PDB ID: 5n8pA, normal Z-score of 1.52, Cov of 0.97, TM-score of 0.916, and RMSD of 1.04 Å).

The generated CTL and HTL 3D models were both further refined by ModRefiner to repair any gaps, followed by GalaxyRefine refinement. The refinement by ModRefiner showed TM-scores of 0.9189 and 0.9498 for the CTL and HTL models, respectively; because these values are close to 1, the initial and refined models were structurally similar. After refinement, the RMSDs for the CTL and HTL models with respect to the initial model were 3.367 Å and 2.318 Å, respectively. Further, both the CTL and HTL MEV models were refined with GalaxyRefine, and model 1 was chosen based on the best scoring parameters. The CTL MEV model refinement output model (Ramachandran favored 83.6%, GDT-HA 0.9371, RMSD 0.459, MolProbity 2.539, clash score 23.2, and poor rotamers 1.8) and the HTL MEV model refinement output model (Ramachandran favored 87.7%, GDT-HA 0.9552, RMSD 0.402, MolProbity 2.537, clash score 27.9, and poor rotamers 1.6) show that well-refined and acceptable models were generated for both the MEVs. After refinement, all the mentioned parameters were found to be significantly improved in comparison to the initial CTL and HTL MEV models (Supplementary Table S3, Multimedia Appendix 1).

#### Validation of the Refined Models of the CTL and HTL MEVs

Both the CTL and HTL models were analyzed with the RAMPAGE analysis tool after refinement. The refined CTL MEV model was found to have 85.8% residues in the favored region, 11.3% residues in the allowed region, and only 3.0% residues in the outlier region; meanwhile, the refined HTL MEV model was found to have 88.9% residues in the favored region, 8.9% residues in the allowed region, and only 2.2% residues in the outlier region (Supplementary Figure S5, Multimedia Appendix 1).

#### Linear and Discontinuous B-cell Epitope Prediction From the MEVs

Linear and discontinuous B-cell epitope prediction was performed to identify potential linear and discontinuous epitopes in the refined 3D models of the CTL and HTL MEVs utilizing the ElliPro tool available on the IEDB server. The screening revealed that the CTL MEV carries 17 linear and 2 potential discontinuous B cell epitopes and the HTL MEV carries 17 linear and 4 potential discontinuous epitopes. The wide range of the PI scores of the linear and discontinuous epitopes in the CTL and HTL MEVs show the high potential of the epitopes to cause humoral immune response (PI scores: CTL MEV linear and discontinuous B cell epitopes: 0.511-0.828 and 0.664-0.767, respectively; HTL MEV linear and discontinuous B cell epitopes: 0.518-0.831 and 0.53-0.776, respectively) (Supplementary Tables S4, S5, S6, and S7, Multimedia Appendix 1).

### Molecular Interaction Analysis of the MEVs With Immunological Receptors

#### Molecular Docking Studies of the MEVs With TLR3

**Figure 4:**
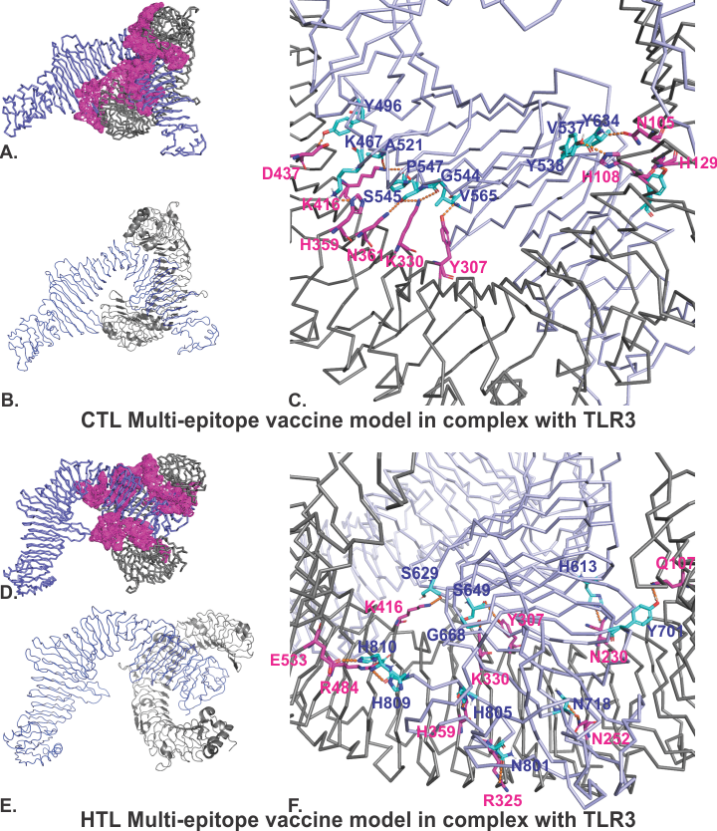
Molecular docking studies of the CTL and HTL MEVs with TLR3. (A), (D): The docking complexes of CTL-TLR3 and HTL-TLR3 with patches on the TLR3 receptor indicating the region of binding sites calculated by PDBsum [60]. (C), (F): Detailed molecular interactions between the binding site residues of the CTL and HTL MEVs and TRL3 (CTL, HTL: cyan; TLR3: magenta). Hydrogen bond formation is shown by orange dotted lines. (B), (E): B-factors of the docked MEVs to the TLR3 receptor. The presentation is in VIBGYOR color, with blue showing a low B-factor and red showing a high B-factor. Most of the MEV regions are blue, showing low B-factors; this indicates the formation of stable complexes with the TLR3 receptor. CTL, cytotoxic T lymphocyte. HTL, helper T lymphocyte. TLR3, toll-like receptor 3.

The refined models of both the CTL and HTL MEVs were further studied for their molecular interactions with the ectodomain of human TLR3. Therefore, molecular docking of the CTL and HTL MEV models with the TLR3 crystal structure model (PDB ID: 2A0Z) was performed utilizing the PatchDock tool. The generated docking conformations with the highest scores of 20776 and 20350 for the CTL and HTL MEVs, respectively, were chosen for further study. The highest docking score indicates the best geometric shape complementarity fitting conformation of the MEV and the TLR3 receptor as predicted by the PatchDock tool. Both the CTL and HTL MEVs fit into the ectodomain region of TLR3 after docking, involving numerous molecular interactions with active site residues of the TLR3 cavity region (Figure 4A, C, D, and F). As shown in Figure 4A and 4D, an entire patch of the TLR3 cavity surface is involved in the molecular interactions with the MEVs, favoring the formation of molecular complexes between the MEVs and the TLR3 ectodomain cavity. Paticular residues involved in this interaction are shown in Fig 4C and 4F (CTL:TLR3: Y496:D437, K467:H359, A521:K416, P547:K416, S545:N361, G544:K330, V565:Y307, Y538:H129, V537:N105, Y634:H108. HTL:TLR3: S629:K416, S649:Y307, G668:K330, H810:E533, H809:R484, H805:H359, N801:R325, H613:N230, N252:N718, Y701:Q107). The CTL and HTL MEVs showed the formation of multiple hydrogen bonds within the ectodomain cavity region of TLR3.

B-factor analysis of the MEV-TLR3 complexes was also performed. The B-factor indicates the displacement of the atomic positions from an average (mean) value, as in, the more flexible the atom, the larger its displacement from the mean position (mean-squares displacement) (Figure 4B, 4D). PDBsum [60] was used to calculate patches on the TLR3 receptor indicating the region of binding sites. The B-factor analysis of the CTL and HTL MEVs bound to the TLR3 receptor shows that most of the regions of the MEVs bound to TLR3 are stable. The B-factor analysis is represented by a VIBGYOR color presentation, where blue represents a low B-factor and red represents a high B-factor (Figure 4B, 4D). These results suggest tendencies toward stable complex formation for both the CTL and HTL MEVs with the ectodomain of the human TLR3 receptor.

#### Molecular Dynamics Simulation Study of the Complexes of the MEVs with TLR3

**Figure 5:**
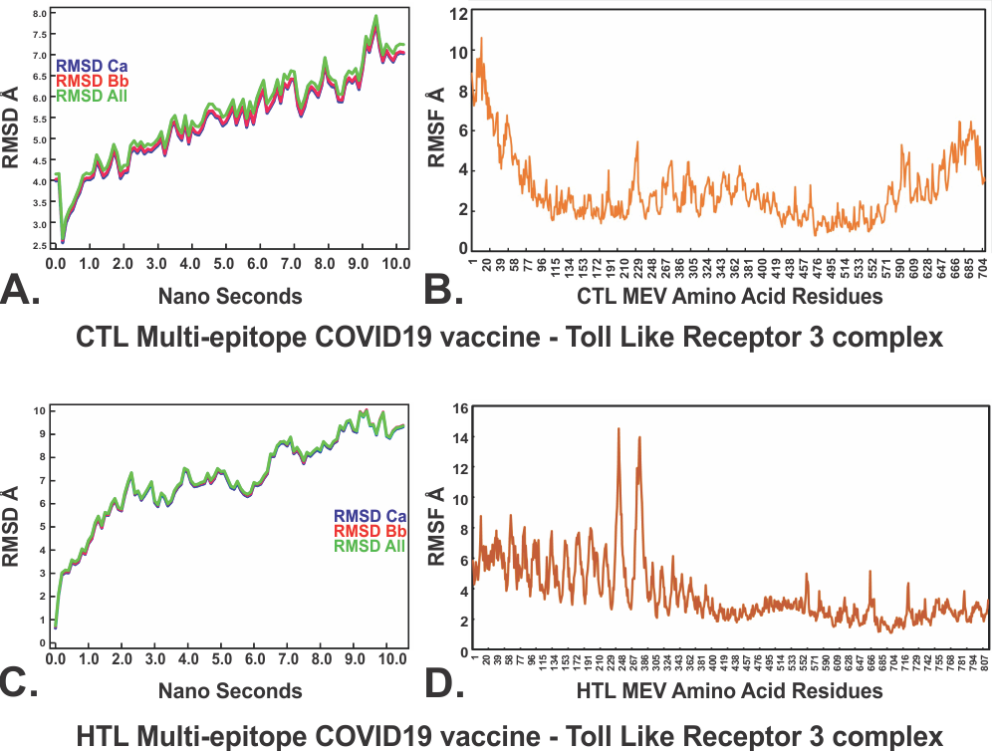
Molecular dynamics simulations of the CTL and HTL MEVs with TLR3. (A), (C): Root mean square deviations for the Cα, backbone, and all atoms for the CTL MEV-TLR3 complex and the HTL MEV-TLR3 complex. (B), (D): Root mean square fluctuations of all the amino acid residues of the CTL MEV and the HTL MEV in complex with the TLR3 immune receptor. Å: angstroms. COVID-19: coronavirus disease. CTL: cytotoxic T lymphocyte. HTL: helper T lymphocyte. MEV: multiepitope vaccine. TL3: toll-like receptor 3. RMSD: root mean square deviation. RMSD Ca: root mean square deviation for the alpha carbon atoms. RMSD Bb: root mean square deviation for the backbone atoms. RMSD All: root mean square deviation for all atoms. RMSF: root mean square fluctuation.

Both the complexes CTL-TLR3 and HTL-TLR3 were further subjected to molecular dynamics simulation analysis to investigate the stability of the molecular interactions involved. Both the MEV-TLR3 complexes showed very convincing and reasonably stable RMSD values for the C_α_, backbone, and all atoms (CTL-TLR3 complex: approximately 4-7.5 Å; HTL-TLR3 complex: approximately 3.0-9.8 Å) which stabilized toward the end (Figure 5A and 5C). The RMSDs of both complexes remained in the abovementioned RMSD range for a given time window of 10 ns at reasonably invariable temperature (approximately 278 K) and pressure (approximately 1 atm). The molecular docking and molecular dynamics simulation studies of all the MEV-TLR complexes indicate tendencies toward stable complex formation. Almost all the AA residues of the CTL and HTL MEVs complexed with TLR3 showed RMSFs in an acceptable range (approximately 2-6 Å) (Figure 5B and 5D). These results indicate that both the CTL-TLR3 and HTL-TLR3 complexes are stable, with acceptable molecular interaction tendencies.

### In Silico Analysis of cDNA of the MEVs for Cloning and Expression Potency in a Mammalian Host Cell Line

cDNA optimized for CTL and HTL expression in a mammalian (human) host cell line was generated using the Java Codon Adaptation Tool. Further, the generated optimized cDNAs for both the MEVs were analyzed using the GenScript Rare Codon Analysis Tool. The analysis revealed that the codon-optimized cDNAs of both the CTL and HTL MEVs have crucial and favorable compositions for high-level expression in a mammalian cell line (CTL MEV: GC content 70.40%, CAI score 1.00, and 0% tandem rare codons; HTL MEV: GC content 69.26%, CAI score 1.00, and 0% tandem rare codons). Ideally, the GC content of cDNA should be 30%-70%; a CAI score that indicates the possibility of cDNA expression in a chosen expression system should be between 0.8 and 1.0; and the tandem rare codon frequency that indicates the presence of low-frequency codons in cDNA should be <30%. Tandem rare codons may hinder proper expression of the cDNA or even interrupt the translational machinery of the chosen expression system. Therefore, as per the GenScript Rare Codon analysis, the cDNAs of both the MEVs satisfy all the mentioned parameters and are predicted to have high expression in the mammalian (human) host cell line.

## Discussion

### Principal Findings

In the present study, we have reported the design of CTL and HTL multiepitope-based vaccine candidates against SARS-CoV-2 infection. These MEVs are composed of multiple CTL and HTL epitopes with truncated Ov-ASP-1 as an adjuvant at the N termini of both the MEVs. To design the abovementioned MEVs, we screened potential CTL and HTL epitopes from the entire proteome of the SARS-CoV-2 coronavirus. The screened epitopes showed potential due to their low IC_50_ values (nM) for HLA interaction, high immunogenicity, nontoxicity, favorable TAP cavity interaction, high conservancy, and high percentile rankings (determined using the IEDB MHC-I Binding Predictions and MHC-II Binding Predictions tools). Furthermore, the population coverage of the shortlisted 38 CTL and 33 HLT epitopes and their HLA allele binders was analyzed; the results were very satisfying, with a total world population coverage of 96.10%. Moreover, 12 B cell epitopes with lengths of 4-20 AAs were screened that showed full or partial overlap with the shortlisted CTL and HTL epitopes. All the shortlisted epitopes were highly conserved, with a conservancy range between 97.08% and 100%; at the same time, all the epitopes were nontoxic. All the shortlisted CTL, HTL, and B cell epitopes were also shown to overlap with each other, which further indicated their highly immunogenic nature. The overlapping epitopes of CTL and HTL were chosen for further analysis of their molecular interactions with HLA alleles and the TAP cavity. Molecular interaction analysis of the chosen overlapping epitopes with their respective HAL allele binders showed very favorable results. Similarly, the molecular interaction analysis of the CTL epitopes within the TAP cavity showed very favorable results for the smooth passage of the epitopes through the cavity from the cytoplasmic end (C terminal) to the ER lumen end (N terminal) of the transmembrane transporter. Further, the two MEVs were designed and modeled utilizing a flexible linker (GGGGS). The chosen adjuvant (truncated Ov-ASP-1) was linked at the N terminal of both the MEVs using a rigid linker (EAAAK). Modeling and further refinement of both the MEVs was performed, and highly sterically acceptable models were generated. The molecular weights of both the MEVs were also very acceptable for expression in suitable systems (CTL MEV: 72.62 kilodaltons, HTL MEV: 82.80 kDa). Further, both the MEVs were shown to contain 20 INFγ-inducing positive epitopes. Both the MEVs were also analyzed to contain numerous linear (CTL: 17, HTL: 17) and discontinuous (CTL: 2, HTL: 4) B cell epitopes. Both the MEVs were analyzed and found to be nonallergenic but antigenic in nature.

Furthermore, both the CTL and HTL MEVs were analyzed for their molecular interactions with the immune receptor TLR3. TLRs act as sentinels for the human immune system; therefore, favorable and stable interactions of both the MEVs with TLR3 are essential. In our study, we confirmed the stable interactions of both the CTL and HTL MEVs with the TLR3 receptor. Molecular docking studies revealed that numerous residues of both MEVs are involved in the formation of polar contacts with TLR3 receptor AA residues. Furthermore, the molecular dynamics studies confirmed stable molecular interactions between both MEVs and TLR3 based on the acceptable RMSDs for the backbones of both the CTL-MEV-TLR3 and HTL-MEV-TLR3 complexes.

Moreover, both MEVs were shown to have very favorable expression in vitro. We analyzed the codon-biased cDNAs for both the CTL and HTL MEVs for the mammalian (human) cell line expression system and found very acceptable CG contents and CAIs as well as 0% tandem rare codons. Therefore, both the designed MEVs can be expressed in the chosen expression system and further tested in vivo as potential vaccine candidates against SARS-CoV-2 infection.

### Conclusion

We have designed and proposed two MEVs derived from multiple CTL and HTL epitopes against SARS-CoV-2 (COVID-19). The chosen CTL and HTL epitopes show significant sequence overlap with screened linear B cell epitopes. The shortlisted CTL and HTL epitopes were used to design CTL and HTL MEVs. Tertiary models of both the generated CTL and HTL MEVs were shown to contain potential linear and discontinuous B cell epitopes as well as potential INFγ epitopes. Therefore, the designed MEVs are predicted to be capable of eliciting humoral and cellular immune responses. Because Ov-ASP-1 binds to APCs and triggers pro-inflammatory cytokine production via TLR3, truncated Ov-ASP-1 was used as an adjuvant at the N termini of both the CTL and HTL MEV models. The molecular interactions of the chosen overlapping clustering epitopes with their respective HLA allele binders were validated by molecular docking studies. The molecular interactions of the chosen CTL epitopes with the TAP transporter cavity were also analyzed. Analysis of the average world population coverage by both the shortlisted CTL and HTL epitopes combined revealed coverage of 96.10% of the world population. The molecular interaction analysis of both the CTL and HTL MEVs with the immunoreceptor TLR3 showed very convincing structural fitting of the MEVs into the ectodomain of the TLR3 cavity. This result was further confirmed by molecular dynamics simulation studies of both the CTL-MEV-TLR3 and HTL-MEV-TLR3 complexes, indicating tendencies toward stable molecular complex formation of both MEVs with TLR3. cDNAs for both MEVs were generated considering codon-biasing for expression in a mammalian (human) host cell line. Both cDNAs were optimized with respect to their GC content and zero tandem rare codons to increase their possibility of high expression in the mammalian host cell line (human). Therefore, for further studies, both the designed CTL and HTL MEVs could be cloned, expressed, and tested for in vivo validation and animal trials as potential vaccine candidates against SARS-CoV-2 infection.

## Appendix

Multimedia Appendix 1 Supplementary material.

## Abbreviations

AA: amino acid
Cα: alpha carbon
cDNA: complementary DNA
COVID-19: coronavirus disease
CTL: cytotoxic T lymphocyte
E protein: envelope protein
ER: endoplasmic reticulum
HLA: human leukocyte antigen
HTL: helper T lymphocyte
IC50: half maximal inhibitory concentration
IEDB: Immune Epitope Database
IFNγ: interferon gamma
MERCI: Motif-EmeRging and with Classes-Identification
MEV: multiepitope vaccine
MHC: major histocompatibility complex
M protein: membrane protein
NCBI: National Center for Biology Information
N protein: nucleocapsid protein
ORF: Open Reading Frame
Ov-ASP-1: Onchocerca volvulus activation-associated secreted protein-1
PDB: Protein Data Bank
PI: protrusion index
RMSD: root mean square deviation
RMSF: root mean square fluctuation
SARS-CoV-2: severe acute respiratory syndrome coronavirus 2
S protein: surface protein
TAP: transporter associated with antigen processing
TLR: toll-like receptor
TLR3: toll-like receptor 3
TrEMBL: Translated European Molecular Biology Laboratory
VIBGYOR: violet-indigo-blue-green-yellow-orange-red
YASARA: Yet Another Scientific Artificial Reality Application
