# Supplementary material for: Structural Basis for Designing Multiepitope Vaccines Against COVID-19 Infection: In Silico Vaccine Design and Validation
Source: JMIR Bioinform Biotechnol. 2020 Jun 19;1(1):e19371. doi: 10.2196/19371 (PMC7370533; doi:10.2196/19371)
Supplement: Multimedia Appendix 1 [file bioinform_v1i1e19371_app1.pdf]

SUPPLEMENTARY:

Structural basis for designing multi-epitope vaccines against novel coronavirus 19 infection, the ongoing pandemic emergency: an in silico approach

Sukrit Srivastava[†a,b], Sonia Verma[c], Mohit Kamthania[d], Rupinder Kaur[e], Ruchi Kiran Badyal[f], Ajay Kumar Saxena[b], Ho-Joon Shin[g], Michael Kolbe[h,I], Kailash C Pandey[c]

a Infection Biology Group, Department of Biotechnology, Institute of Bio-Medical Education and Research, Mangalayatan University, 202146 Aligarh, India.
b Molecular Medicine Lab., School of Life Science, Jawaharlal Nehru University, 110067 New Delhi, India.
c Parasite-Host Biology Group, Protein Biochemistry & Engineering Lab, ICMR-National Institute of Malaria Research, New Delhi, India
d Department of Biotechnology, Institute of Applied Medicines and Research, 201206 Ghaziabad, India.
e Department of Chemistry, Guru Nanak Dev University, Amritsar, India
f Department of economics, Mangalayata University, India
g Department of Microbiology,School of Medicine, Ajou University, South Korea
h Department for Structural Infection Biology, Centre for Structural Systems Biology (CSSB) & Helmholtz-Centre for Infection Research, Notkestraße 85, 22607 Hamburg, Germany.
i Faculty of Mathematics, Informatics and Natural Sciences, University of Hamburg, Rothenbaumchaussee 19, 20148 Hamburg, Germany.

[†]Correspondence should be addressed to S.S. (sukrit.srivastav@mangalayatan.edu.in; srivastav.sukrit@gmail.com)

Supplementary table S1. Homology modeling for HLA alleles. Tertiary structures of HLA alleles were modeled by homology modeling using SwissModel server. Templates were chosen with the highest sequence identity. Generated models with acceptable QMEAN values were chosen for further studies.

S.No	HLA Class I Allele	Template used for modeling	% sequence identity	QMEAN
1	A*31:01	6o9b.1.A	94.29%	0.73
2	A*11:01	6o9b.1.A	97.50%	1.14
S.No	HLA Class II Allele	Template used for modeling	% sequence identity	QMEAN
3	DRB3*01:01	4is6.1.B	90.10%	(-)1.84
4	DRB1*09:01	1bx2.1.B	87.43%	(-)0.46

Supplementary table S2. INF-γ epitopes from CTL and HTL MEVs. INF-γ inducing (POSITIVE) epitopes from CTL and HTL MEVs were screened by using “Motif and SVM hybrid” (MERCI & SVM) approach.

CLT Epitopes also predicted to be IFN-gamma epitopes					
S.No	Start-END	Sequence	Method	Result	Score
1	67-82	RWANQCIFGHSPRQQ	MERCI	POSITIVE	2
2	68-83	WANQCIFGHSPRQQR	MERCI	POSITIVE	2
3	69-84	ANQCIFGHSPRQQRE	MERCI	POSITIVE	2
4	70-85	NQCIFGHSPRQQREG	MERCI	POSITIVE	2
5	71-86	QCIFGHSPRQQREGV	MERCI	POSITIVE	2
6	83-98	EGVGENVYAYWSSVS	MERCI	POSITIVE	1
7	84-99	GVGENVYAYWSSVSV	MERCI	POSITIVE	2
8	120-135	LYENNPSNNMTWKVA	MERCI	POSITIVE	1
9	121-136	YENNPSNNMTWKVAG	MERCI	POSITIVE	1
10	122-137	ENNPSNNMTWKVAGQ	MERCI	POSITIVE	2
11	413-428	CTDDNALAYYGGGGS	MERCI	POSITIVE	1
12	428-443	TDDNALAYYGGGGSM	MERCI	POSITIVE	1
13	429-444	DDNALAYYGGGGSMY	MERCI	POSITIVE	1
14	439-454	GGGGSSIINNTVYTK	MERCI	POSITIVE	1
15	440-455	GGGSSIINNTVYTKG	MERCI	POSITIVE	2
16	441-456	GGSSIINNTVYTKGG	MERCI	POSITIVE	3
17	442-457	GSSIINNTVYTKGGG	MERCI	POSITIVE	3
18	443-458	SSIINNTVYTKGGGG	MERCI	POSITIVE	3
19	444-459	SIINNTVYTKGGGGS	MERCI	POSITIVE	3
20	445-460	IINNTVYTKGGGGSL	MERCI	POSITIVE	3
HLT Epitopes also predicted to be IFN-gamma epitopes					
S.No	Start-END	Sequence	Method	Result	Score
1	67-82	RWANQCIFGHSPRQQ	MERCI	POSITIVE	2
2	68-83	WANQCIFGHSPRQQR	MERCI	POSITIVE	2
3	69-84	ANQCIFGHSPRQQRE	MERCI	POSITIVE	2
4	70-85	NQCIFGHSPRQQREG	MERCI	POSITIVE	2
5	71-86	QCIFGHSPRQQREGV	MERCI	POSITIVE	2
6	83-98	EGVGENVYAYWSSVS	MERCI	POSITIVE	1
7	84-99	GVGENVYAYWSSVSV	MERCI	POSITIVE	2
8	120-135	LYENNPSNNMTWKVA	MERCI	POSITIVE	1
9	121-136	YENNPSNNMTWKVAG	MERCI	POSITIVE	1
10	122-137	ENNPSNNMTWKVAGQ	MERCI	POSITIVE	2
11	585-600	GGGGSFAWWTAFVTN	MERCI	POSITIVE	1
12	586-601	GGGSFAWWTAFVTNV	MERCI	POSITIVE	1
13	587-602	GGSFAWWTAFVTNVN	MERCI	POSITIVE	1
14	588-603	GSFAWWTAFVTNVNA	MERCI	POSITIVE	1
15	589-604	SFAWWTAFVTNVNAS	MERCI	POSITIVE	1
16	560-575	FAWWTAFVTNVNASS	MERCI	POSITIVE	1
17	561-576	AWWTAFVTNVNASSG	MERCI	POSITIVE	1
18	806-821	GGGSCTQHQPYVVDD	MERCI	POSITIVE	1
19	807-822	GGSCTQHQPYVVDDP	MERCI	POSITIVE	1
20	808-823	GSCTQHQPYVVDDPC	MERCI	POSITIVE	1

Supplementary table S3. Refinement models of CTL and HTL MEVs. Both the CTL and HTL MEVs models were refined by GalaxyWEB server. After refinement, in particular the Rama favored residues increased significantly.

Galaxy Refinement for CTL MEV						
Model	GDT-HA	RMSD	Mol Probity	Clash score	Poor rotamers	Rama favored
Initial	1.0000	0.000	3.749	100.5	4.5	75.8
MODEL 1	0.9371	0.459	2.539	23.2	0.4	83.6
Galaxy Refinement for HTL MEV						
Initial	1.0000	0.000	3.554	131.2	2.2	81.3
MODEL 1	0.9552	0.402	2.537	27.9	0.7	87.7

Supplementary table S4. B Cell linear epitopes of CTL MEVs. Linear B Cell epitopes predicted by ElliPro (IEDB) from CTL MEVs.

CTL Multi-Epitope vaccine model linear epitopes					
S.No.	Start	End	Peptide	Number of residues	Score
1	1	97	IVVAVTGYNCPGGKLTALERKKIVGQNNKYRSDLINGKLKNRNGTY MPRGKNMLELTWDCKLESSAQRWANQCIFGHSPRQQREGVGENVYAYWSSV	97	0.828
2	604	704	LRARSVSPKGGGGSFLAFLLFLVGGGGSHFYSKWYIRGGGGSWTAG AAAYYVGGGGSFPNITNLCPFGGGGSNYNYLYRLFRGGGGSNYLYRLFRHHHHHH	101	0.812
3	400	415	LNGSCGSVGGGGSCTD	16	0.693
4	265	277	SSEMVMCGGSLYG	13	0.678
5	422	438	GGGGSCTDDNALAYYGG	17	0.662
6	584	596	FGGGGSRARSVSP	13	0.619
7	219	231	GGGSKPRQKRTAT	13	0.618
8	490	502	WSMATYYGGGGSY	13	0.618
9	294	304	GGGSISNSWLM	11	0.607
10	362	372	FYGGGGSFLFV	11	0.599
11	380	393	GGGSRYFRLTLGVY	14	0.599
12	320	327	KGGGGSQE	8	0.55
13	445	456	GLPWNVVRGGGG	12	0.548
14	99	109	VEGLKKTAGTD	11	0.547
15	342	349	FNVPMEKG	8	0.534
16	521	524	ASFY	4	0.528
17	464	477	VYTKGGGGSLPVNV	14	0.511

Supplementary table S5. B Cell discontinuous epitopes of CTL MEVs. Discontinuous B Cell epitopes predicted by ElliPro (IEDB) from CTL MEVs.

CTL Multi-Epitope vaccine model discontinuous epitopes			
S.No.	Residues	Number of residues	Score
1	I1, V2, V3, A4, V5, T6, G7, Y8, N9, C10, P11, G12, G13, K14, L15, T16, A17, L18, E19, R20, K21, K22, I23, V24, G25, Q26, N27, N28, K29, Y30, R31, S32, D33, L34, I35, N36, G37, K38, L39, K40, N41, R42, N43, G44, T45, Y46, M47, P48, R49, G50, K51, N52, M53, L54, E55, L56, T57, W58, D59, C60, K61, L62, E63, S64, S65, A66, Q67, R68, W69, A70, N71, Q72, C73, I74, F75, G76, H77, S78, P79, R80, Q81, Q82, R83, E84, G85, V86, G87, E88, N89, V90, Y91, A92, Y93, W94, S95, S96, V97, S98, V99, E100, G101, L102, K103, K104, T105, G107, T108, D109, K112, W114, W115, N125, P126, S127, N128, N129, K133, V134, A135, G136	120	0.767
2	G160, A187, R188, G189, G219, G220, G221, S222, K223, P224, R225, Q226, R228, T229, A230, T231, G246, G247, G248, G249, S250, G264, S265, S266, E267, M268, V269, M270, C271, G272, G273, S274, L275, Y276, G277, G294, G295, G296, S297, I298, S299, N300, S301, W302, L303, M304, K320, G321, G322, G323, G324, S325, Q326, E327, I328, T341, F342, N343, V344, P345, M346, E347, K348, G349, Y363, G364, G365, G366, G367, S368, F369, L370, F371, V372, A374, G380, G381, G382, S383, R384, Y385, F386, R387, L388, T389, L390, G391, V392, L400, N401, G402, S403, C404, G405, S406, V407, G408, G409, G410, G411, S412, C413, T414, D415, G422, G423, G424, G425, S426, C427, T428, D429, D430, N431, A432, L433, A434, Y435, Y436, G445, L446, P447, W448, N449, V450, V451, R452, G453, G454, G455, G456, G469, G470, G471, S472, L473, P474, V475, N476, V477, W490, M492, A493, T494, Y495, Y496, G497, G498, G499, G500, S501, Y502, A521, S522, F523, Y524, I546, P547, Y548, N549, S550, K564, V565, S566, I567, W568, F584, G585, G586, G587, G588, S589, R590, A591, R592, S593, V594, S595, P596, G598, G599, G600, G601, Q603, L604, R605, A606, R607, S608, V609, S610, P611, K612, G613, G614, G615, G616, S617, F618, L619, A620, F621, L622, L623, F624, L625, V626, G627, G628, G629, G630, S631, H632, F633, Y634, S635, K636, W637, Y638, I639, R640, G641, G642, G643, G644, S645, W646, T647, A648, G649, A650, A651, A652, Y653, Y654, V655, G656, G657, G658, G659, S660, F661, P662, N663, I664, T665, N666, L667, C668, P669, F670, G671, G672, G673, G674, S675, N676, Y677, N678, Y679, L680, Y681, R682, L683, F684, G686, G687, G688, G689, S690, N691, Y692, L693, Y694, R695, R698, H699, H700, H701, H702, H703, H704	282	0.664

Supplementary table S6. B Cell linear epitopes of HTL MEVs. Linear B Cell epitopes predicted by ElliPro (IEDB) from HTL MEVs.

HTL Multi-Epitope vaccine model linear epitopes					
S.No.	Start	End	Peptide	Number of residues	Score
1	691	810	INVFAFPFTIYSLLGGGGSKTQSLLIVNNATNVVGGGGSLLIVNNATNVVIK VCGGGGSQSLLIVNNATNVVIKGGGGSSLLIVNNATNVVIKVGGGGSTQ SLLIVNNATNVVIHHHHHH	120	0.831
2	1	138	IVVAVTGYNCPGGKLTALERKKIVGQNNKYRSDLINGKLKNRNGTYMPR GKNMLELTWDCKLESSAQRWANQCIFGHSPRQQREGVGENVYAYWSS VSVEGLKKTAGTDAGKSWWSKLPKLYENNPSNNMTWKVAGQG	138	0.777
3	452	468	VSIWNLDYIINLIGGGG	17	0.713
4	323	333	FVGGGGSESPF	11	0.69
5	474	489	WNLDYIINLIIGGGGS	16	0.685
6	434	444	TVYSHLLLVAA	11	0.679
7	543	551	LYGGGGSCF	9	0.651
8	416	424	TIPIQASLP	9	0.63
9	278	287	SAFFGMSGGG	10	0.608
10	351	360	ILASFSASTS	10	0.597
11	496	508	WNLDYIINLGGGG	13	0.586
12	399	404	PAQYEL	6	0.572
13	378	383	SAPPAQ	6	0.565
14	673	684	FAFPFTIYSLLL	12	0.556
15	515	527	ALITLATCELGGG	13	0.544
16	570	573	LCFL	4	0.54
17	655	660	DDPCPI	6	0.518

Supplementary table S7. B Cell discontinuous epitopes of HTL MEVs. Discontinuous B Cell epitopes predicted by ElliPro (IEDB) from HTL MEVs.

HTL Multi-Epitope vaccine model discontinuous epitopes			
S. No.	Residues	Number of residues	Score
1	C639, P640, I641, D655, D656, P657, C658, P659, I660, H661, F662, F673, A674, F675, P676, F677, T678, I679, Y680, S681, L682, L683, L684, G687, G688, I691, N692, V693, F694, A695, F696, P697, F698, T699, I700, Y701, S702, L703, L704, G705, G706, G707, G708, S709, K710, T711, Q712, S713, L714, L715, I716, V717, N718, N719, A720, T721, N722, V723, V724, G725, G726, G727, G728, S729, L730, L731, I732, V733, N734, N735, A736, T737, N738, V739, V740, I741, K742, V743, C744, G745, G746, G747, G748, S749, Q750, S751, L752, L753, I754, V755, N756, N757, A758, T759, N760, V761, V762, I763, K764, G765, G766, G767, G768, S769, S770, L771, L772, I773, V774, N775, N776, A777, T778, N779, V780, V781, I782, K783, V784, G785, G786, G787, G788, S789, T790, Q791, S792, L793, L794, I795, V796, N797, N798, A799, T800, N801, V802, V803, I804, H805, H806, H807, H808, H809, H810	145	0.776
2	I1, V2, V3, A4, V5, T6, G7, Y8, N9, C10, P11, G12, G13, K14, L15, T16, A17, L18, E19, R20, K21, K22, I23, V24, G25, Q26, N27, N28, K29, Y30, R31, S32, D33, L34, I35, N36, G37, K38, L39, K40, N41, R42, N43, G44, T45, Y46, M47, P48, R49, G50, K51, N52, M53, L54, E55, L56, T57, W58, D59, C60, K61, L62, E63, S64, S65, A66, Q67, R68, W69, A70, N71, Q72, C73, I74, F75, G76, H77, S78, P79, R80, Q81, Q82, R83, E84, G85, V86, G87, E88, N89, V90, Y91, A92, Y93, W94, S95, S96, V97, S98, V99, E100, G101, L102, K103, K104, T105, A106, G107, T108, D109, A110, G111, K112, S113, W114, W115, S116, K117, L118, P119, K120, L121, Y122, E123, N124, N125, P126, S127, N128, N129, M130, T131, W132, K133, V134, A135, G136, Q137, G138, E145, A146, A147, A148, L150, F152, A154, V156, L163, A164, G165, G168, S169	151	0.748
3	S278, A279, F280, F281, G282, M283, S284, G285, G286, G287, F303, G304, G305, A322, F323, V324, G325, G326, G327, G328, S329, E330, S331, P332, F333, I350, I351, L352, A353, S354, F355, S356, A357, S358, T359, S360, S378, A379, P380, P381, A382, Q383, P399, A400, Q401, Y402, E403, L404, G405, G406, G407, T416, I417, P418, I419, Q420, A421, S422, L423, P424, T434, V435, Y436, S437, H438, L439, L440, L441, V442, A444, G446, V452, S453, I454, W455, N456, L457, D458, Y459, I460, I461, N462, L463, I464, G465, G466, G467, G468, W474, N475, L476, D477, Y478, I479, I480, N481, L482, I483, I484, G485, G486, G487, G488, S489, I495, W496, N497, L498, D499, Y500, I501, I502, N503, L504, G505, G506, G507, G508, A515, L516, I517, T518, A520, T521, C522, E523, L538, A539, L543, Y544, G545, G546, G547, G548, S549, C550, F551, L570, C571, F572, L573, F596, L597, L598, F599, P615, Y616, V617, V618, D619	150	0.609
4	L524, G525, G526, G527, G528	5	0.53

Supplementary table S8. Shortlisted high scoring CTL epitopes (MHC-I Binding Predictions). Selected high scoring CTL epitopes and their respective HLA alleles binders predicted by "MHC-I Binding Predictions" IEDB tool. *In-silico* analysis has shown all the selected epitopes to be non-toxic (Non-Toxin) as well as they show significant conservancy and high immunogenicity.

SARS-CoV-2 CTL epitopes (MHC-I Binding Predictions)									
S.No.	Proteins	Peptide	Allele	Length	Immunogenicity	Conservancy	Toxicity	Method used	Percentile Rank
1	E Protein	FLAFVVFLL	A*02:01	9	0.30188	99.59% (480/482)	Non-Toxin	Consensus (ann/comblib_sidney2008/smm)	0.2
2	E Protein	FLAFVVFLL	A*02:03	9	0.30188	99.59% (480/482)	Non-Toxin	Consensus (ann/smm)	0.25
3	E Protein	FLAFVVFLLV	A*02:01	10	0.30526	99.59% (480/482)	Non-Toxin	Consensus (ann/smm)	0.15
4	E Protein	FLAFVVFLLV	A*02:03	10	0.30526	99.59% (480/482)	Non-Toxin	Consensus (ann/smm)	0.23
5	E Protein	LAFVVFLLV	B*51:01	9	0.2141	99.59% (480/482)	Non-Toxin	Consensus (ann/comblib_sidney2008/smm)	0.2
6	E Protein	LVKPSFYVY	B*15:01	9	-0.11106	99.59% (480/482)	Non-Toxin	Consensus (ann/comblib_sidney2008/smm)	0.2
7	E Protein	RVKNLNSSR	A*31:01	9	-0.32968	99.59% (480/482)	Non-Toxin	Consensus (ann/smm)	0.16
8	E Protein	SLVKPSFYVY	B*15:01	10	-0.2443	99.59% (480/482)	Non-Toxin	Consensus (ann/smm)	0.28
9	E Protein	SVLLFLAFV	A*02:06	9	0.19022	99.59% (480/482)	Non-Toxin	Consensus (ann/smm)	0.33
10	E Protein	TLAILTALR	A*68:01	9	0.1989	99.17% (478/482)	Non-Toxin	Consensus (ann/smm)	0.21
11	E Protein	VLLFLAFVV	A*02:01	9	0.26315	99.59% (480/482)	Non-Toxin	Consensus (ann/comblib_sidney2008/smm)	0.3
12	E Protein	VSLVKPSFY	A*30:02	9	-0.25372	99.59% (480/482)	Non-Toxin	Consensus (ann/smm)	0.33
13	M Protein	FIASFRLFAR	A*68:01	10	0.12185	99.79% (476/477)	Non-Toxin	Consensus (ann/smm)	0.12
14	M Protein	LIFLWLLWPV	A*02:06	10	0.40176	98.32% (469/477)	Non-Toxin	Consensus (ann/smm)	0.08
15	M Protein	LSYFIASFR	A*68:01	9	0.21181	97.90% (467/477)	Non-Toxin	Consensus (ann/smm)	0.11
16	M Protein	MWLSYFIASF	A*23:01	10	0.00197	97.69% (466/477)	Non-Toxin	Consensus (ann/smm)	0.11
17	M Protein	MWLSYFIASFR	A*68:01	11	0.03554	97.69% (466/477)	Non-Toxin	ann	0.09
18	M Protein	MWLSYFIASFR	A*33:01	11	0.03554	97.69% (466/477)	Non-Toxin	ann	0.1
19	M Protein	QWNLVIGFLF	A*23:01	10	0.28076	99.16% (473/477)	Non-Toxin	Consensus (ann/smm)	0.12
20	M Protein	RFLYIIKLIF	A*23:01	10	0.11728	98.74% (471/477)	Non-Toxin	Consensus (ann/smm)	0.12
21	M Protein	RTRSMWSF	A*32:01	8	-0.25178	99.58% (475/477)	Non-Toxin	ann	0.1
22	N Protein	AQFAPSASAF	B*15:01	10	-0.17446	97.59% (486/498)	Non-Toxin	Consensus (ann/smm)	0.16
23	N Protein	AQFAPSASAFF	B*15:01	11	-0.11074	97.59% (486/498)	Non-Toxin	ann	0.12
24	N Protein	FTALTQHGK	A*68:01	9	-0.0226	99.40% (495/498)	Non-Toxin	Consensus (ann/smm)	0.18
25	N Protein	KMKDLSPR	A*31:01	8	-0.22357	99.60% (496/498)	Non-Toxin	ann	0.15
26	N Protein	KTFPPTEPK	A*11:01	9	0.1306	96.99% (483/498)	Non-Toxin	Consensus (ann/smm)	0.11
27	N Protein	KTFPPTEPK	A*30:01	9	0.1306	96.99% (483/498)	Non-Toxin	Consensus (ann/comblib_sidney2008/smm)	0.2
28	N Protein	RQKRTATKAY	B*15:01	10	-0.06462	97.79% (487/498)	Non-Toxin	Consensus (ann/smm)	0.2
29	N Protein	SSPDDQIGYY	A*01:01	10	0.07924	99.40% (495/498)	Non-Toxin	Consensus (ann/smm)	0.2
30	ORF-1ab	ASFYYVWKSY	A*30:02	10	0.00073	99.78% (455/456)	Non-Toxin	Consensus (ann/smm)	0.07
31	ORF-1ab	CLAYYFMRFRR	A*33:01	11	0.12614	100% (456/456)	Non-Toxin	ann	0.07
32	ORF-1ab	ETISLAGSY	A*26:01	9	-0.1653	100% (456/456)	Non-Toxin	Consensus (ann/smm)	0.1
33	ORF-1ab	IPLMYKGLPW	B*53:01	10	-0.37784	100% (456/456)	Non-Toxin	Consensus (ann/smm)	0.07
34	ORF-1ab	IPLMYKGLPW	B*53:01	10	-0.37784	100% (456/456)	Non-Toxin	Consensus (ann/smm)	0.07
35	ORF-1ab	KMNYQVNGY	A*30:02	9	-0.06542	100% (456/456)	Non-Toxin	Consensus (ann/smm)	0.07

36	ORF-1ab	KMNYQVNGY	A*30:02	9	-0.06542	100% (456/456)	Non-Toxin	Consensus (ann/smm)	0.07
37	ORF-1ab	STNVTIATY	A*30:02	9	0.25822	99.34% (453/456)	Non-Toxin	Consensus (ann/smm)	0.09
38	ORF-1ab	YAYLRKHFSM	B*08:01	10	-0.13937	100% (456/456)	Non-Toxin	Consensus (ann/smm)	0.07
39	ORF3a	FMRIFTIGTV	A*02:03	10	0.47908	99.37% (478/481)	Non-Toxin	Consensus (ann/smm)	0.17
40	ORF3a	FTIGTVTLK	A*68:01	9	0.18024	99.37% (478/481)	Non-Toxin	Consensus (ann/smm)	0.16
41	ORF3a	HVTFFIYNK	A*68:01	9	0.36278	99.79% (480/481)	Non-Toxin	Consensus (ann/smm)	0.16
42	ORF3a	IIMRLWLCWK	A*03:01	10	0.27346	96.67% (465/481)	Non-Toxin	Consensus (ann/smm)	0.13
43	ORF3a	VEHVTFFIY	B*44:03	9	0.3766	99.58% (479/481)	Non-Toxin	Consensus (ann/smm)	0.18
44	ORF3a	YFLQSINFVR	A*33:01	10	-0.03483	96.88% (466/481)	Non-Toxin	Consensus (ann/smm)	0.2
45	ORF6	AEILLIIM	B*40:01	8	0.25884	99.58% (479/481)	Non-Toxin	Consensus (ann/smm)	0.41
46	ORF6	FQVTIAEIL	A*02:06	9	0.38115	99.17% (477/481)	Non-Toxin	Consensus (ann/smm)	0.52
47	ORF6	ILLIIMRTFK	A*03:01	10	0.2388	99.58% (479/481)	Non-Toxin	Consensus (ann/smm)	0.17
48	ORF6	IMRTFKVSI	A*32:01	9	-0.09496	99.79% (480/481)	Non-Toxin	Consensus (ann/comblib_sidney2008/smm)	0.44
49	ORF6	KVSIWNLDY	A*30:02	9	0.29343	99.58% (479/481)	Non-Toxin	Consensus (ann/smm)	0.41
50	ORF6	LLIIMRTFK	A*03:01	9	0.156	99.58% (479/481)	Non-Toxin	Consensus (ann/smm)	0.36
51	ORF6	LSKSLTENKY	A*30:02	10	-0.24668	98.75% (475/481)	Non-Toxin	Consensus (ann/smm)	0.48
52	ORF6	SIWNLDYII	A*32:01	9	0.15011	99.58% (479/481)	Non-Toxin	Consensus (ann/comblib_sidney2008/smm)	0.2
53	ORF6	VTIAEILLI	B*58:01	9	0.28951	99.17% (477/481)	Non-Toxin	Consensus (ann/comblib_sidney2008/smm)	0.34
54	ORF7a	CVRGTTVLLK	A*03:01	10	0.14952	99.58% (478/480)	Non-Toxin	Consensus (ann/smm)	0.17
55	ORF7a	EVQELYSPI	A*26:01	9	-0.09723	98.12% (471/480)	Non-Toxin	Consensus (ann/smm)	0.21
56	ORF7a	EVQELYSPIF	A*26:01	10	-0.03858	98.12% (471/480)	Non-Toxin	Consensus (ann/smm)	0.22
57	ORF7a	FALTCFSTQF	B*53:01	10	-0.09369	98.75% (474/480)	Non-Toxin	Consensus (ann/smm)	0.22
58	ORF7a	FLIVAAIVF	B*15:01	9	0.29611	98.96% (475/480)	Non-Toxin	Consensus (ann/comblib_sidney2008/smm)	0.2
59	ORF7a	FLIVAAIVFI	A*02:01	10	0.38946	98.33% (472/480)	Non-Toxin	Consensus (ann/smm)	0.21
60	ORF7a	ITLCFTLKRK	A*03:01	10	-0.06825	97.92% (470/480)	Non-Toxin	Consensus (ann/smm)	0.17
61	ORF7a	QELYSPIFLI	B*44:02	10	0.03838	98.33% (472/480)	Non-Toxin	Consensus (ann/smm)	0.22
62	ORF7a	QELYSPIFLI	B*44:03	10	0.03838	98.33% (472/480)	Non-Toxin	Consensus (ann/smm)	0.25
63	ORF7a	RSVSPKLFI	B*58:01	9	-0.30783	98.33% (472/480)	Non-Toxin	Consensus (ann/comblib_sidney2008/smm)	0.2
64	ORF7b	DFYLCFLAF	A*23:01	9	0.05884	98.31% (232/236)	Non-Toxin	Consensus (ann/smm)	0.29
65	ORF7b	FLAFLLFLVL	A*02:01	10	0.23386	98.31% (232/236)	Non-Toxin	Consensus (ann/smm)	0.26
66	ORF7b	FLLFLVLIML	A*02:01	10	0.14288	99.58% (235/236)	Non-Toxin	Consensus (ann/smm)	0.3
67	ORF7b	FYLCFLAFLL	A*23:01	10	0.1745	98.31% (232/236)	Non-Toxin	Consensus (ann/smm)	0.13
68	ORF7b	FYLCFLAFLL	A*24:02	10	0.1745	98.31% (232/236)	Non-Toxin	Consensus (ann/smm)	0.28
69	ORF7b	IELSLIDFY	B*44:03	9	0.03153	99.58% (235/236)	Non-Toxin	Consensus (ann/smm)	0.19
70	ORF7b	LVLIMLIIFW	B*53:01	10	0.25452	99.58% (235/236)	Non-Toxin	Consensus (ann/smm)	0.32
71	ORF7b	MIELSLIDFY	A*01:01	10	0.06184	99.58% (235/236)	Non-Toxin	Consensus (ann/smm)	0.35
72	ORF7b	MLIIFWFSL	A*02:01	9	0.50177	99.58% (235/236)	Non-Toxin	Consensus (ann/comblib_sidney2008/smm)	0.2
73	ORF7b	SLIDFYLCFL	A*02:01	10	0.18838	98.31% (232/236)	Non-Toxin	Consensus (ann/smm)	0.14
74	ORF7b	YLCFLAFLL	A*02:01	9	0.21865	98.31% (232/236)	Non-Toxin	Consensus (ann/comblib_sidney2008/smm)	0.3
75	ORF8	CPIHFYSKW	B*53:01	9	-0.07935	99.38% (477/480)	Non-Toxin	Consensus (ann/comblib_sidney2008/smm)	0.2
76	ORF8	CPIHFYSKWY	B*53:01	10	-0.02216	99.38% (477/480)	Non-Toxin	Consensus (ann/smm)	0.2
77	ORF8	CSFYEDFLEY	A*01:01	10	0.31272	99.79% (479/480)	Non-Toxin	Consensus (ann/smm)	0.24
78	ORF8	DFLEYHDVR	A*33:01	9	0.16684	99.58% (478/480)	Non-Toxin	Consensus (ann/smm)	0.13
79	ORF8	FTINCQEPK	A*68:01	9	-0.04683	100.00% (480/480)	Non-Toxin	Consensus (ann/smm)	0.18
80	ORF8	GARKSAPLI	A*30:01	9	-0.34031	99.17% (476/480)	Non-Toxin	Consensus (ann/comblib_sidney2008/smm)	0.3

81	ORF8	GIITTVAAF	B*15:01	9	0.2148	99.17% (476/480)	Non-Toxin	Consensus (ann/comblib_sidney2008/smm)	0.3
82	ORF8	GSLVVRCSFY	A*30:02	10	0.00657	100.00% (480/480)	Non-Toxin	Consensus (ann/smm)	0.24
83	ORF8	IQYIDIGNY	A*30:02	9	0.30442	99.38% (477/480)	Non-Toxin	Consensus (ann/smm)	0.12
84	ORF8	LGIITTVAAF	B*15:01	10	0.34746	99.17% (476/480)	Non-Toxin	Consensus (ann/smm)	0.31
85	ORF8	LQSCTQHQPY	B*15:01	10	-0.25674	96.04% (461/480)	Non-Toxin	Consensus (ann/smm)	0.17
86	ORF8	NYTVSCLPF	A*23:01	9	-0.17355	51.46% (247/480)	Non-Toxin	Consensus (ann/smm)	0.23
87	ORF8	QSCTQHQPY	A*01:01	9	-0.16503	96.04% (461/480)	Non-Toxin	Consensus (ann/smm)	0.28
88	ORF10	FAFPFTIYSL	A*02:06	10	0.20414	99.79% (478/479)	Non-Toxin	Consensus (ann/smm)	0.48
89	ORF10	FPFTIYSL	B*51:01	8	0.06356	100.00% (479/479)	Non-Toxin	Consensus (ann/smm)	0.39
90	ORF10	FPFTIYSLL	B*51:01	9	0.05708	100.00% (479/479)	Non-Toxin	Consensus (ann/comblib_sidney2008/smm)	0.3
91	ORF10	FPFTIYSLLL	B*53:01	10	0.03149	100.00% (479/479)	Non-Toxin	Consensus (ann/smm)	0.13
92	ORF10	FPFTIYSLLL	B*51:01	10	0.03149	100.00% (479/479)	Non-Toxin	Consensus (ann/smm)	0.14
93	ORF10	FTIYSLLLCR	A*68:01	10	-0.18372	100.00% (479/479)	Non-Toxin	Consensus (ann/smm)	0.12
94	ORF10	FTIYSLLLCR	A*33:01	10	-0.18372	100.00% (479/479)	Non-Toxin	Consensus (ann/smm)	0.35
95	ORF10	MGYINVFAFPF	A*23:01	11	0.40977	99.37% (476/479)	Non-Toxin	Consensus (ann/smm)	0.41
96	ORF10	TIYSLLLCR	A*68:01	9	-0.22977	100.00% (479/479)	Non-Toxin	Consensus (ann/smm)	0.47
97	ORF10	YINVFAFPF	B*53:01	9	0.28259	99.37% (476/479)	Non-Toxin	Consensus (ann/comblib_sidney2008/smm)	0.35
98	ORF10	YINVFAFPF	A*32:01	9	0.28259	99.37% (476/479)	Non-Toxin	Consensus (ann/comblib_sidney2008/smm)	0.4
99	S Protein	FVFLVLLPLV	A*02:06	10	0.02996	98.94% (467/472)	Non-Toxin	Consensus (ann/smm)	0.1
100	S Protein	GNYNYLYRLFR	A*33:01	11	0.08205	98.52% (465/472)	Non-Toxin	ann	0.11
101	S Protein	KSFTVEKGIY	A*30:02	10	0.11812	99.36% (469/472)	Non-Toxin	Consensus (ann/smm)	0.11

Supplementary table S9. Shortlisted high scoring CTL epitopes (MHC-I Processing Predictions). Selected high scoring CTL epitopes and their respective HLA alleles binders predicted by "MHC-I Processing Predictions" IEDB tool. The screening gives detailed and combined scoring "Total score" for Proteasomal cleavage/TAP transport/MHC class I combined. *In-silico* analysis have shown all the selected epitopes to be non-toxic (Non-Toxin) as well as they show significant conservancy with high immunogenicity.

SARS-CoV-2 CTL epitopes (MHC-I Processing Predictions)													
S.No.	Proteins	Allele	Peptide Length	Peptide	Immunogenicity	Conservancy	Toxicity	Proteasome Score	TAP Score	MHC Score	Processing Score	Total Score	MHC IC50[nM]
1	E Protein	B*15:01	10	SLVKPSFYVY	-0.2443	99.59% (480/482)	Non-Toxin	1.51	1.36	-1.39	2.86	1.47	24.6
2	E Protein	B*15:01	9	LVKPSFYVY	-0.11106	99.59% (480/482)	Non-Toxin	1.51	1.35	-1.42	2.86	1.44	26.3
3	E Protein	A*30:02	9	LVKPSFYVY	-0.11106	99.59% (480/482)	Non-Toxin	1.51	1.35	-1.61	2.86	1.25	40.4
4	E Protein	A*02:01	9	FLAFVVFLL	0.30188	99.59% (480/482)	Non-Toxin	1.45	0.41	-0.81	1.86	1.05	6.5
5	E Protein	B*15:01	9	LTALRLCAY	0.01886	99.17% (478/482)	Non-Toxin	1.42	1.27	-1.69	2.69	1	49.5
6	M Protein	A*30:02	9	ATSRTLSYY	-0.11604	98.95% (472/477)	Non-Toxin	1.26	1.34	-1.12	2.6	1.48	13.3
7	M Protein	A*11:01	9	ATSRTLSYY	-0.11604	98.95% (472/477)	Non-Toxin	1.26	1.34	-1.52	2.6	1.08	32.9
8	M Protein	B*08:01	10	FARTRSMWSF	-0.12986	99.58% (475/477)	Non-Toxin	1.41	1.12	-1.34	2.53	1.19	22.1
9	M Protein	A*02:01	10	FLWLLWPVTL	0.31272	98.53% (470/477)	Non-Toxin	1.85	0.46	-1.16	2.31	1.16	14.3
10	M Protein	A*68:01	9	LSYFIASFR	0.21181	97.90% (467/477)	Non-Toxin	0.84	0.72	-0.46	1.56	1.1	2.9
11	M Protein	A*23:01	11	LSYFIASFRLF	0.2706	97.90% (467/477)	Non-Toxin	1.25	1.18	-1.36	2.43	1.07	23

12	M Protein	A*23:01	10	MWLSYFIASF	0.00197	97.69% (466/477)	Non-Toxin	1.38	1.26	-0.91	2.63	1.73	8.1
13	M Protein	A*24:02	10	MWLSYFIASF	0.00197	97.69% (466/477)	Non-Toxin	1.38	1.26	-1.27	2.63	1.36	18.7
14	M Protein	B*15:01	10	MWLSYFIASF	0.00197	97.69% (466/477)	Non-Toxin	1.38	1.26	-1.33	2.63	1.3	21.6
15	M Protein	A*23:01	10	RFLYIIKLIF	0.11728	98.74% (471/477)	Non-Toxin	1.25	1.35	-1.33	2.6	1.27	21.3
16	M Protein	A*30:01	8	RTRSMWSF	-0.25178	99.58% (475/477)	Non-Toxin	1.41	1.28	-1.51	2.69	1.18	32.7
17	M Protein	A*30:02	9	SGFAAYSRY	0.00261	98.32% (469/477)	Non-Toxin	1.53	1.17	-1.4	2.69	1.3	25
18	M Protein	B*15:01	10	SQRVAGDSGF	0.0305	98.53% (470/477)	Non-Toxin	1.33	1.24	-1.35	2.58	1.23	22.4
19	M Protein	A*23:01	9	SYFIASFRL	0.18333	98.74% (471/477)	Non-Toxin	1.45	0.62	-0.99	2.08	1.09	9.8
20	M Protein	B*35:01	9	VATSRTLSY	-0.17295	98.95% (472/477)	Non-Toxin	1.34	1.31	-1.38	2.65	1.27	23.9
21	M Protein	A*30:02	9	YSRYRIGNY	0.21358	98.53% (470/477)	Non-Toxin	1.36	1.37	-1.61	2.73	1.12	40.6
22	N Protein	B*15:01	10	AQFAPSASAF	-0.17446	97.59% (486/498)	Non-Toxin	1.23	1.25	-0.7	2.48	1.78	5
23	N Protein	B*15:01	11	AQFAPSASAFF	-0.11074	97.59% (486/498)	Non-Toxin	1.3	1.25	-1.46	2.55	1.08	29
24	N Protein	A*30:02	10	KDLSPRWYFY	0.14332	99.60% (496/498)	Non-Toxin	1.58	1.21	-1.68	2.79	1.1	48.3
25	N Protein	A*30:02	10	KMKDLSPRWY	-0.05692	99.60% (496/498)	Non-Toxin	1.31	1.35	-1.56	2.66	1.1	36.2
26	N Protein	A*68:02	9	NTASWFTAL	0.22775	99.60% (496/498)	Non-Toxin	1.42	0.49	-0.79	1.91	1.12	6.2
27	N Protein	B*15:01	10	RQKRTATKAY	-0.06462	97.79% (487/498)	Non-Toxin	1.51	1.34	-1.55	2.85	1.3	35.5
28	N Protein	A*01:01	10	SSPDDQIGYY	0.07924	99.40% (495/498)	Non-Toxin	1.24	1.36	-1.16	2.6	1.45	14.3
29	ORF1ab	A*01:01	10	ACTDDNALAY	0.10055	99.78% (455/456)	Non-Toxin	1.49	1.33	-0.82	2.82	2	6.6
30	ORF1ab	A*01:01	9	CTDDNALAY	0.07355	99.78% (455/456)	Non-Toxin	1.49	1.23	-0.45	2.72	2.27	2.8
31	ORF1ab	A*26:01	9	ETISLAGSY	-0.1653	100% (456/456)	Non-Toxin	1.4	1.21	-0.53	2.61	2.08	3.4
32	ORF1ab	B*35:01	9	FAVDAAKAY	-0.04849	98.90% (451/456)	Non-Toxin	1.73	1.35	-0.46	3.08	2.62	2.9
33	ORF1ab	B*15:01	9	ILMTARTVY	0.12576	100% (456/456)	Non-Toxin	1.65	1.34	-1.1	2.99	1.89	12.5
34	ORF1ab	B*15:01	10	LILMTARTVY	0.0012	100% (456/456)	Non-Toxin	1.65	1.36	-1.23	3.01	1.78	16.9
35	ORF1ab	B*15:01	10	LMSNLGMPSY	-0.30933	99.56% (454/456)	Non-Toxin	1.55	1.34	-1	2.9	1.89	10.1
36	ORF1ab	B*35:01	9	LPSLATVAY	0.06748	98.02% (447/456)	Non-Toxin	1.47	1.15	-0.34	2.62	2.28	2.2
37	ORF1ab	A*32:01	9	RMYIFFASF	0.29328	99.12% (452/456)	Non-Toxin	1.32	1.36	-0.82	2.68	1.86	6.6
38	ORF1ab	A*30:02	10	RMYIFFASFY	0.32633	99.12% (452/456)	Non-Toxin	1.28	1.53	-0.97	2.81	1.83	9.4
39	ORF1ab	A*03:01	10	RMYIFFASFY	0.32633	99.12% (452/456)	Non-Toxin	1.28	1.53	-1	2.81	1.8	10.1
40	ORF1ab	B*44:03	9	SEFSSLPSY	-0.40603	99.34% (453/456)	Non-Toxin	1.49	1.35	-1.03	2.84	1.82	10.6
41	ORF1ab	B*15:01	9	VMYMGTLSY	-0.21438	97.58% (445/456)	Non-Toxin	1.3	1.5	-0.8	2.79	1.99	6.3
42	ORF1ab	A*03:01	9	VMYMGTLSY	-0.21438	97.58% (445/456)	Non-Toxin	1.3	1.5	-0.98	2.79	1.82	9.5
43	ORF3a	B*44:03	11	AGLEAPFLYLY	0.21841	96.67% (465/481)	Non-Toxin	1.51	1.29	-1.63	2.8	1.17	42.9
44	ORF3a	A*23:01	10	LVYFLQSINF	-0.05419	96.88% (466/481)	Non-Toxin	1.3	1.31	-1.39	2.61	1.23	24.3
45	ORF6	A*30:02	9	KVSIWNLDY	0.29343	99.58% (479/481)	Non-Toxin	1.21	1.33	-1.46	2.54	1.08	28.8
46	ORF7a	B*35:01	9	CPDGVKHVY	-0.07008	98.54% (473/480)	Non-Toxin	1.43	1.11	-1.34	2.54	1.2	22
47	ORF7b	A*02:01	9	IIFWFSLEL	0.2683	99.58% (235/236)	Non-Toxin	1.65	0.55	-1.2	2.21	1.01	15.7
48	ORF7b	A*02:01	10	SLIDFYLCFL	0.18838	98.31% (232/236)	Non-Toxin	1.7	0.5	-0.74	2.2	1.46	5.5
49	ORF7b	A*02:03	10	SLIDFYLCFL	0.18838	98.31% (232/236)	Non-Toxin	1.7	0.5	-0.79	2.2	1.41	6.2
50	ORF7b	A*02:06	10	SLIDFYLCFL	0.18838	98.31% (232/236)	Non-Toxin	1.7	0.5	-0.93	2.2	1.27	8.6
51	ORF8	B*15:01	9	GIITTVAAF	0.2148	99.17% (476/480)	Non-Toxin	1.27	1.13	-1.26	2.4	1.14	18.1
52	ORF8	A*33:01	9	HFYSKWYIR	-0.09452	99.38% (477/480)	Non-Toxin	1.08	0.76	-0.68	1.84	1.16	4.8
53	ORF8	A*31:01	9	HFYSKWYIR	-0.09452	99.38% (477/480)	Non-Toxin	1.08	0.76	-0.79	1.84	1.05	6.2
54	ORF8	B*15:01	10	LGIITTVAAF	0.34746	99.17% (476/480)	Non-Toxin	1.27	0.97	-1.1	2.24	1.14	12.7
55	ORF10	A*68:01	10	NVFAFPFTIY	0.40129	99.58% (477/479)	Non-Toxin	1.47	1.4	-1.84	2.86	1.02	69.7

56	S Protein	B*35:01	9	CVADYSVLY	-0.09595	100.00% (472/472)	Non-Toxin	1.51	1.38	-1.39	2.89	1.5	24.4
57	S Protein	B*35:01	9	FAMQMAYRF	-0.28061	100.00% (472/472)	Non-Toxin	1.45	1.05	-0.8	2.5	1.7	6.3
58	S Protein	B*58:01	10	KRSFIEDLLF	0.29624	99.36% (469/472)	Non-Toxin	1.23	1.26	-1.11	2.49	1.37	12.9
59	S Protein	A*23:01	10	KWPWYIWLGF	0.56424	99.58% (470/472)	Non-Toxin	1.26	1.25	-1.04	2.51	1.47	11
60	S Protein	B*35:01	11	LQIPFAMQMAY	-0.22124	100.00% (472/472)	Non-Toxin	1.42	1.4	-1.25	2.82	1.57	17.8
61	S Protein	A*01:01	9	LTDEMIAQY	0.02757	99.58% (470/472)	Non-Toxin	1.21	1.21	-0.72	2.42	1.71	5.2
62	S Protein	B*35:01	10	QIPFAMQMAY	-0.25308	100.00% (472/472)	Non-Toxin	1.42	1.35	-1.16	2.77	1.61	14.5
63	S Protein	A*30:02	9	RISNCVADY	-0.02787	100.00% (472/472)	Non-Toxin	1.16	1.47	-1.26	2.63	1.37	18.2
64	S Protein	B*58:01	9	RSFIEDLLF	0.27446	99.58% (470/472)	Non-Toxin	1.23	1.32	-0.72	2.54	1.82	5.3
65	S Protein	B*15:01	10	RVYSTGSNVF	-0.23394	100.00% (472/472)	Non-Toxin	1.51	1.32	-1.02	2.83	1.81	10.5
66	S Protein	B*35:01	9	SANNCTFEY	0.13273	98.94% (467/472)	Non-Toxin	1.18	1.3	-1.11	2.48	1.37	12.8
67	S Protein	A*30:02	10	YTNSFTRGVY	0.08467	99.58% (470/472)	Non-Toxin	1.36	1.28	-1.26	2.64	1.38	18.2

Supplementary table S10. Shortlisted high scoring HTL epitopes (MHC-II Binding Predictions). Selected high "Percentile rank" HTL epitopes with their respective HLA class II alleles binders predicted by the "MHC-II Binding Predictions" tool of IEDB are listed. *In-silico* analysis has shown all the selected epitopes to be non-toxic (Non-Toxin) as well as they show significant conservancy.

SARS-CoV-2 HTL epitopes (MHC-II Binding Predictions)							
S.No	Proteins	Allele	Peptide	Conservancy	Toxicity	Method used	Percentile Rank
1	E Protein	DPA1*03:01/DPB1*04:02	FLAFVVFLLVTLAIL	99.59% (480/482)	Non-Toxin	Consensus (comb.lib./smm/nn)	0.06
2	E Protein	DPA1*03:01/DPB1*04:02	LFLAFVVFLLVTLAI	99.59% (480/482)	Non-Toxin	Consensus (comb.lib./smm/nn)	0.03
3	E Protein	DPA1*01:03/DPB1*02:01	LFLAFVVFLLVTLAI	99.59% (480/482)	Non-Toxin	Consensus (comb.lib./smm/nn)	0.04
4	E Protein	DPA1*01/DPB1*04:01	LFLAFVVFLLVTLAI	99.59% (480/482)	Non-Toxin	Consensus (comb.lib./smm)	0.08
5	E Protein	DPA1*02:01/DPB1*01:01	LFLAFVVFLLVTLAI	99.59% (480/482)	Non-Toxin	Consensus (comb.lib./smm/nn)	0.1
6	E Protein	DPA1*01:03/DPB1*02:01	NSVLLFLAFVVFLLV	99.59% (480/482)	Non-Toxin	Consensus (comb.lib./smm/nn)	0.04
7	E Protein	DPA1*03:01/DPB1*04:02	NSVLLFLAFVVFLLV	99.59% (480/482)	Non-Toxin	Consensus (comb.lib./smm/nn)	0.06
8	E Protein	DPA1*01/DPB1*04:01	NSVLLFLAFVVFLLV	99.59% (480/482)	Non-Toxin	Consensus (comb.lib./smm)	0.07
9	E Protein	DPA1*02:01/DPB1*01:01	NSVLLFLAFVVFLLV	99.59% (480/482)	Non-Toxin	Consensus (comb.lib./smm/nn)	0.1
10	E Protein	DPA1*02:01/DPB1*01:01	VNSVLLFLAFVVFLL	99.59% (480/482)	Non-Toxin	Consensus (comb.lib./smm/nn)	0.1
11	M Protein	DQA1*01:01/DQB1*05:01	IKLIFLWLLWPVTLA	98.11% (468/477)	Non-Toxin	Consensus (comb.lib./smm/nn)	0.07
12	M Protein	DQA1*01:01/DQB1*05:01	KLIFLWLLWPVTLAC	98.11% (468/477)	Non-Toxin	Consensus (comb.lib./smm/nn)	0.11
13	M Protein	DRB1*09:01	RTLSYYKLGASQRVA	99.16% (473/477)	Non-Toxin	Consensus (comb.lib./smm/nn)	0.06
14	M Protein	DRB1*01:01	RTLSYYKLGASQRVA	99.16% (473/477)	Non-Toxin	Consensus (comb.lib./smm/nn)	0.67
15	M Protein	DRB1*09:01	SRTLSYYKLGASQRV	99.16% (473/477)	Non-Toxin	Consensus (comb.lib./smm/nn)	0.07
16	M Protein	DRB1*09:01	SYYKLGASQRVAGDS	98.74% (471/477)	Non-Toxin	Consensus (comb.lib./smm/nn)	0.48
17	M Protein	DRB1*09:01	TLSYYKLGASQRVAG	98.95% (472/477)	Non-Toxin	Consensus (comb.lib./smm/nn)	0.07
18	M Protein	DRB1*01:01	TLSYYKLGASQRVAG	98.95% (472/477)	Non-Toxin	Consensus (comb.lib./smm/nn)	0.67
19	M Protein	DPA1*01:03/DPB1*02:01	VGLMWLSYFIASFRL	97.48% (465/477)	Non-Toxin	Consensus (comb.lib./smm/nn)	0.07
20	N Protein	DQA1*01:02/DQB1*06:02	ANNAAIVLQLPQGTT	97.59% (486/498)	Non-Toxin	Consensus (comb.lib./smm/nn)	0.2
21	N Protein	DRB1*11:01	DQIGYYRRATRRIRG	99.40% (495/498)	Non-Toxin	Consensus (smm/nn/sturniolo)	0.42
22	N Protein	DRB5*01:01	DQIGYYRRATRRIRG	99.40% (495/498)	Non-Toxin	Consensus (smm/nn/sturniolo)	0.58

23	N Protein	DQA1*01:02/DQB1*06:02	GTRNPANNAAIVLQL	97.59% (486/498)	Non-Toxin	Consensus (comb.lib./smm/nn)	0.05
24	N Protein	DRB1*07:01	GTWLTYTGAIKLDDK	97.59% (486/498)	Non-Toxin	Consensus (comb.lib./smm/nn)	0.58
25	N Protein	DRB1*11:01	GYYRRATRRIRGGDG	99.40% (495/498)	Non-Toxin	Consensus (smm/nn/sturniolo)	0.42
26	N Protein	DRB1*11:01	IGYYRRATRRIRGGD	99.40% (495/498)	Non-Toxin	Consensus (smm/nn/sturniolo)	0.42
27	N Protein	DQA1*01:02/DQB1*06:02	NNAAIVLQLPQGTTL	97.59% (486/498)	Non-Toxin	Consensus (comb.lib./smm/nn)	0.42
28	N Protein	DQA1*01:02/DQB1*06:02	NPANNAAIVLQLPQG	97.59% (486/498)	Non-Toxin	Consensus (comb.lib./smm/nn)	0.03
29	N Protein	DQA1*01:02/DQB1*06:02	PANNAAIVLQLPQGT	97.59% (486/498)	Non-Toxin	Consensus (comb.lib./smm/nn)	0.04
30	N Protein	DRB1*09:01	QIAQFAPSASAFFGM	97.39% (485/498)	Non-Toxin	Consensus (comb.lib./smm/nn)	0.01
31	N Protein	DRB1*11:01	QIGYYRRATRRIRGG	99.40% (495/498)	Non-Toxin	Consensus (smm/nn/sturniolo)	0.39
32	N Protein	DRB5*01:01	QIGYYRRATRRIRGG	99.40% (495/498)	Non-Toxin	Consensus (smm/nn/sturniolo)	0.58
33	N Protein	DQA1*01:02/DQB1*06:02	RNPANNAAIVLQLPQ	97.59% (486/498)	Non-Toxin	Consensus (comb.lib./smm/nn)	0.04
34	N Protein	DRB1*07:01	SGTWLTYTGAIKLDD	97.39% (485/498)	Non-Toxin	Consensus (comb.lib./smm/nn)	0.58
35	N Protein	DRB1*07:01	TPSGTWLTYTGAIKL	97.39% (485/498)	Non-Toxin	Consensus (comb.lib./smm/nn)	0.58
36	N Protein	DQA1*01:02/DQB1*06:02	TRNPANNAAIVLQLP	97.39% (485/498)	Non-Toxin	Consensus (comb.lib./smm/nn)	0.04
37	N Protein	DRB1*07:01	TWLTYTGAIKLDDKD	97.59% (486/498)	Non-Toxin	Consensus (comb.lib./smm/nn)	0.58
38	ORF1ab	DPA1*01:03/DPB1*02:01	WFLAYILFTRFFYVL	100% (456/456)	Non-Toxin	Consensus (comb.lib./smm/nn)	0.01
39	ORF1ab	DPA1*01:03/DPB1*02:01	FLAYILFTRFFYVLG	100% (456/456)	Non-Toxin	Consensus (comb.lib./smm/nn)	0.01
40	ORF1ab	DPA1*01:03/DPB1*02:01	LAYILFTRFFYVLGL	100% (456/456)	Non-Toxin	Consensus (comb.lib./smm/nn)	0.01
41	ORF1ab	DPA1*01:03/DPB1*02:01	AYILFTRFFYVLGLA	100% (456/456)	Non-Toxin	Consensus (comb.lib./smm/nn)	0.01
42	ORF1ab	DRB1*15:01	AMPNMLRIMASLVLA	100% (456/456)	Non-Toxin	Consensus (smm/nn/sturniolo)	0.01
43	ORF1ab	DRB1*15:01	MPNMLRIMASLVLAR	100% (456/456)	Non-Toxin	Consensus (smm/nn/sturniolo)	0.01
44	ORF1ab	DRB1*15:01	PNMLRIMASLVLARK	100% (456/456)	Non-Toxin	Consensus (smm/nn/sturniolo)	0.01
45	ORF1ab	DRB1*15:01	NMLRIMASLVLARKH	100% (456/456)	Non-Toxin	Consensus (smm/nn/sturniolo)	0.01
46	ORF1ab	DRB3*02:02	FAWWTAFVTNVNASS	98.46% (449/456)	Non-Toxin	NetMHCIIpan	0.01
47	ORF1ab	DPA1*01:03/DPB1*02:01	AAIMQLFFSYFAVHF	100% (456/456)	Non-Toxin	Consensus (comb.lib./smm/nn)	0.05
48	ORF1ab	DQA1*01:02/DQB1*06:02	AFASEAARVVRSIFS	99.56% (454/456)	Non-Toxin	Consensus (comb.lib./smm/nn)	0.08
49	ORF1ab	DPA1*01:03/DPB1*02:01	AIMQLFFSYFAVHFI	100% (456/456)	Non-Toxin	Consensus (comb.lib./smm/nn)	0.05
50	ORF1ab	DRB3*02:02	ANYIFWRNTNPIQLS	98.90% (451/456)	Non-Toxin	NetMHCIIpan	0.05
51	ORF1ab	DQA1*05:01/DQB1*03:01	ASIVAGGIVAIVVTC	100% (456/456)	Non-Toxin	Consensus (comb.lib./smm/nn)	0.03
52	ORF1ab	DRB3*02:02	AWWTAFVTNVNASSS	98.46% (449/456)	Non-Toxin	NetMHCIIpan	0.01
53	ORF1ab	DQA1*05:01/DQB1*03:01	DISASIVAGGIVAIV	100% (456/456)	Non-Toxin	Consensus (comb.lib./smm/nn)	0.03
54	ORF1ab	DPA1*03:01/DPB1*04:02	EETKFLTENLLLYID	99.78% (455/456)	Non-Toxin	Consensus (comb.lib./smm/nn)	0.03
55	ORF1ab	DQA1*05:01/DQB1*02:01	EIDFLELAMDEFIER	99.12% (452/456)	Non-Toxin	Consensus (comb.lib./smm/nn)	0.04
56	ORF1ab	DPA1*03:01/DPB1*04:02	ETKFLTENLLLYIDI	99.78% (455/456)	Non-Toxin	Consensus (comb.lib./smm/nn)	0.03
57	ORF1ab	DQA1*01:01/DQB1*05:01	FISNSWLMWLIINLV	100% (456/456)	Non-Toxin	Consensus (comb.lib./smm/nn)	0.05
58	ORF1ab	DRB1*07:01	FSAVGNICYTPSKLI	99.56% (454/456)	Non-Toxin	Consensus (comb.lib./smm/nn)	0.06
59	ORF1ab	DRB1*07:01	FTPLVPFWITIAYII	100% (456/456)	Non-Toxin	Consensus (comb.lib./smm/nn)	0.03
60	ORF1ab	DQA1*01:01/DQB1*05:01	HFISNSWLMWLIINL	100% (456/456)	Non-Toxin	Consensus (comb.lib./smm/nn)	0.05
61	ORF1ab	DQA1*05:01/DQB1*02:01	IDFLELAMDEFIERY	99.34% (453/456)	Non-Toxin	Consensus (comb.lib./smm/nn)	0.05
62	ORF1ab	DQA1*05:01/DQB1*03:01	ISASIVAGGIVAIVV	100% (456/456)	Non-Toxin	Consensus (comb.lib./smm/nn)	0.03
63	ORF1ab	DQA1*01:01/DQB1*05:01	ISNSWLMWLIINLVQ	100% (456/456)	Non-Toxin	Consensus (comb.lib./smm/nn)	0.07
64	ORF1ab	DPA1*03:01/DPB1*04:02	KLINIIIWFLLLSVC	97.80% (446/456)	Non-Toxin	Consensus (comb.lib./smm/nn)	0.09
65	ORF1ab	DPA1*02:01/DPB1*05:01	KVTLVFLFVAAIFYL	99.78% (455/456)	Non-Toxin	Consensus (comb.lib./smm/nn)	0.04

66	ORF1ab	DPA1*01:03/DPB1*02:01	LAAIMQLFFSYFAVH	100% (456/456)	Non-Toxin	Consensus (comb.lib./smm/nn)	0.07
67	ORF1ab	DPA1*03:01/DPB1*04:02	LEETKFLTENLLLYI	99.78% (455/456)	Non-Toxin	Consensus (comb.lib./smm/nn)	0.03
68	ORF1ab	DPA1*03:01/DPB1*04:02	LINIIIWFLLLSVCL	97.80% (446/456)	Non-Toxin	Consensus (comb.lib./smm/nn)	0.09
69	ORF1ab	DRB1*07:01	LVPFWITIAYIICIS	100% (456/456)	Non-Toxin	Consensus (comb.lib./smm/nn)	0.03
70	ORF1ab	DQA1*05:01/DQB1*02:01	MEIDFLELAMDEFIE	99.12% (452/456)	Non-Toxin	Consensus (comb.lib./smm/nn)	0.04
71	ORF1ab	DPA1*02:01/DPB1*14:01	MNLKYAISAKNRART	99.78% (455/456)	Non-Toxin	NetMHCIIpan	0.08
72	ORF1ab	DPA1*01:03/DPB1*02:01	MQLFFSYFAVHFISN	100% (456/456)	Non-Toxin	Consensus (comb.lib./smm/nn)	0.05
73	ORF1ab	DPA1*01:03/DPB1*02:01	MYIFFASFYYVWKSY	99.12% (452/456)	Non-Toxin	Consensus (comb.lib./smm/nn)	0.04
74	ORF1ab	DRB1*11:01	NEFYAYLRKHFSMMI	100% (456/456)	Non-Toxin	Consensus (smm/nn/sturniolo)	0.02
75	ORF1ab	DRB3*02:02	NYIFWRNTNPIQLSS	98.90% (451/456)	Non-Toxin	NetMHCIIpan	0.04
76	ORF1ab	DRB1*07:01	PLVPFWITIAYIICI	100% (456/456)	Non-Toxin	Consensus (comb.lib./smm/nn)	0.02
77	ORF1ab	DQA1*05:01/DQB1*02:01	QMEIDFLELAMDEFI	99.12% (452/456)	Non-Toxin	Consensus (comb.lib./smm/nn)	0.03
78	ORF1ab	DPA1*02:01/DPB1*14:01	QMNLKYAISAKNRAR	99.78% (455/456)	Non-Toxin	NetMHCIIpan	0.07
79	ORF1ab	DRB1*01:01	QQESPFVMMSAPPAQ	99.78% (455/456)	Non-Toxin	Consensus (comb.lib./smm/nn)	0.1
80	ORF1ab	DQA1*01:01/DQB1*05:01	QSTQWSLFFFLYENA	92.10% (420/456)	Non-Toxin	Consensus (comb.lib./smm/nn)	0.1
81	ORF1ab	DPA1*01:03/DPB1*02:01	QWSLFFFLYENAFLP	92.10% (420/456)	Non-Toxin	Consensus (comb.lib./smm/nn)	0.04
82	ORF1ab	DPA1*01:03/DPB1*02:01	RMYIFFASFYYVWKS	99.12% (452/456)	Non-Toxin	Consensus (comb.lib./smm/nn)	0.04
83	ORF1ab	DQA1*05:01/DQB1*03:01	SASIVAGGIVAIVVT	100% (456/456)	Non-Toxin	Consensus (comb.lib./smm/nn)	0.03
84	ORF1ab	DRB1*07:01	SAVGNICYTPSKLIE	99.56% (454/456)	Non-Toxin	Consensus (comb.lib./smm/nn)	0.09
85	ORF1ab	DQA1*05:01/DQB1*03:01	SIVAGGIVAIVVTCL	100% (456/456)	Non-Toxin	Consensus (comb.lib./smm/nn)	0.03
86	ORF1ab	DPA1*03:01/DPB1*04:02	SKLINIIIWFLLLSV	97.80% (446/456)	Non-Toxin	Consensus (comb.lib./smm/nn)	0.09
87	ORF1ab	DQA1*01:01/DQB1*05:01	SNSWLMWLIINLVQM	100% (456/456)	Non-Toxin	Consensus (comb.lib./smm/nn)	0.07
88	ORF1ab	DQA1*05:01/DQB1*02:01	SQMEIDFLELAMDEF	99.12% (452/456)	Non-Toxin	Consensus (comb.lib./smm/nn)	0.05
89	ORF1ab	DPA1*01:03/DPB1*02:01	STQWSLFFFLYENAF	92.10% (420/456)	Non-Toxin	Consensus (comb.lib./smm/nn)	0.05
90	ORF1ab	DQA1*01:01/DQB1*05:01	STQWSLFFFLYENAF	92.10% (420/456)	Non-Toxin	Consensus (comb.lib./smm/nn)	0.1
91	ORF1ab	DPA1*03:01/DPB1*04:02	TKFLTENLLLYIDIN	99.78% (455/456)	Non-Toxin	Consensus (comb.lib./smm/nn)	0.05
92	ORF1ab	DPA1*03:01/DPB1*04:02	TLEETKFLTENLLLY	99.78% (455/456)	Non-Toxin	Consensus (comb.lib./smm/nn)	0.05
93	ORF1ab	DRB1*07:01	TPLVPFWITIAYIIC	100% (456/456)	Non-Toxin	Consensus (comb.lib./smm/nn)	0.03
94	ORF1ab	DPA1*01:03/DPB1*02:01	TQWSLFFFLYENAFL	92.10% (420/456)	Non-Toxin	Consensus (comb.lib./smm/nn)	0.04
95	ORF1ab	DQA1*01:01/DQB1*05:01	TQWSLFFFLYENAFL	92.10% (420/456)	Non-Toxin	Consensus (comb.lib./smm/nn)	0.09
96	ORF1ab	DRB1*11:01	VNEFYAYLRKHFSMM	100% (456/456)	Non-Toxin	Consensus (smm/nn/sturniolo)	0.05
97	ORF1ab	DRB1*01:01	VQQESPFVMMSAPPA	99.78% (455/456)	Non-Toxin	Consensus (comb.lib./smm/nn)	0.1
98	ORF1ab	DQA1*01:02/DQB1*06:02	YAFASEAARVVRSIF	99.78% (455/456)	Non-Toxin	Consensus (comb.lib./smm/nn)	0.08
99	ORF1ab	DPA1*01:03/DPB1*02:01	YIFFASFYYVWKSYV	99.56% (454/456)	Non-Toxin	Consensus (comb.lib./smm/nn)	0.05
100	ORF1ab	DRB3*02:02	YIFWRNTNPIQLSSY	98.90% (451/456)	Non-Toxin	NetMHCIIpan	0.05
101	ORF1ab	DPA1*01:03/DPB1*02:01	YILFTRFFYVLGLAA	100% (456/456)	Non-Toxin	Consensus (comb.lib./smm/nn)	0.08
102	ORF3a	DPA1*01/DPB1*04:01	APFLYLYALVYFLQS	96.88% (466/481)	Non-Toxin	Consensus (comb.lib./smm)	0.12
103	ORF3a	DPA1*02:01/DPB1*14:01	DFVRATATIPIQASL	99.37% (478/481)	Non-Toxin	NetMHCIIpan	0.12
104	ORF3a	DPA1*01:03/DPB1*02:01	DTGVEHVTFFIYNKI	99.37% (478/481)	Non-Toxin	Consensus (comb.lib./smm/nn)	1.1
105	ORF3a	DPA1*02:01/DPB1*05:01	DTGVEHVTFFIYNKI	99.37% (478/481)	Non-Toxin	Consensus (comb.lib./smm/nn)	1.6
106	ORF3a	DRB1*04:05	FFIYNKIVDEPEEHV	99.58% (479/481)	Non-Toxin	Consensus (smm/nn/sturniolo)	0.94
107	ORF3a	DPA1*01/DPB1*04:01	FLYLYALVYFLQSIN	96.67% (465/481)	Non-Toxin	Consensus (comb.lib./smm)	0.12
108	ORF3a	DPA1*01:03/DPB1*02:01	GVEHVTFFIYNKIVD	99.37% (478/481)	Non-Toxin	Consensus (comb.lib./smm/nn)	1.2

109	ORF3a	DRB1*04:05	HVTFFIYNKIVDEPE	99.58% (479/481)	Non-Toxin	Consensus (smm/nn/sturniolo)	0.84
110	ORF3a	DPA1*01/DPB1*04:01	PFLYLYALVYFLQSI	96.67% (465/481)	Non-Toxin	Consensus (comb.lib./smm)	0.12
111	ORF3a	DRB1*04:05	TFFIYNKIVDEPEEH	99.58% (479/481)	Non-Toxin	Consensus (smm/nn/sturniolo)	0.81
112	ORF3a	DPA1*01:03/DPB1*02:01	TGVEHVTFFIYNKIV	99.37% (478/481)	Non-Toxin	Consensus (comb.lib./smm/nn)	0.93
113	ORF3a	DPA1*02:01/DPB1*05:01	TGVEHVTFFIYNKIV	99.37% (478/481)	Non-Toxin	Consensus (comb.lib./smm/nn)	1.4
114	ORF3a	DRB1*04:05	VTFFIYNKIVDEPEE	99.58% (479/481)	Non-Toxin	Consensus (smm/nn/sturniolo)	0.81
115	ORF3a	DPA1*01/DPB1*04:01	YLYALVYFLQSINFV	96.46% (464/481)	Non-Toxin	Consensus (comb.lib./smm)	0.14
116	ORF6	DRB1*15:01	EILLIIMRTFKVSIW	99.58% (479/481)	Non-Toxin	Consensus (smm/nn/sturniolo)	0.1
117	ORF6	DRB1*15:01	ILLIIMRTFKVSIWN	99.38% (478/481)	Non-Toxin	Consensus (smm/nn/sturniolo)	0.1
118	ORF6	DQA1*01:01/DQB1*05:01	VSIWNLDYIINLIIK	99.38% (478/481)	Non-Toxin	Consensus (comb.lib./smm/nn)	0.05
119	ORF7a	DRB1*01:01	KIILFLALITLATCE	99.58% (478/480)	Non-Toxin	Consensus (comb.lib./smm/nn)	0.16
120	ORF7b	DRB4*01:01	AFLLFLVLIMLIIFW	99.58% (235/236)	Non-Toxin	Consensus (comb.lib./smm/nn)	0.14
121	ORF7b	DPA1*03:01/DPB1*04:02	DFYLCFLAFLLFLVL	98.31% (232/236)	Non-Toxin	Consensus (comb.lib./smm/nn)	0.03
122	ORF7b	DPA1*01:03/DPB1*02:01	DFYLCFLAFLLFLVL	98.31% (232/236)	Non-Toxin	Consensus (comb.lib./smm/nn)	0.08
123	ORF7b	DPA1*01/DPB1*04:01	DFYLCFLAFLLFLVL	98.31% (232/236)	Non-Toxin	Consensus (comb.lib./smm)	0.09
124	ORF7b	DPA1*03:01/DPB1*04:02	FLAFLLFLVLIMLII	97.88% (231/236)	Non-Toxin	Consensus (comb.lib./smm/nn)	0.06
125	ORF7b	DRB4*01:01	FLAFLLFLVLIMLII	97.88% (231/236)	Non-Toxin	Consensus (comb.lib./smm/nn)	0.14
126	ORF7b	DRB4*01:01	FLLFLVLIMLIIFWF	99.58% (235/236)	Non-Toxin	Consensus (comb.lib./smm/nn)	0.19
127	ORF7b	DPA1*03:01/DPB1*04:02	FYLCFLAFLLFLVLI	97.88% (231/236)	Non-Toxin	Consensus (comb.lib./smm/nn)	0.03
128	ORF7b	DPA1*01:03/DPB1*02:01	FYLCFLAFLLFLVLI	97.88% (231/236)	Non-Toxin	Consensus (comb.lib./smm/nn)	0.06
129	ORF7b	DPA1*01/DPB1*04:01	FYLCFLAFLLFLVLI	97.88% (231/236)	Non-Toxin	Consensus (comb.lib./smm)	0.09
130	ORF7b	DPA1*01:03/DPB1*02:01	IDFYLCFLAFLLFLV	98.31% (232/236)	Non-Toxin	Consensus (comb.lib./smm/nn)	0.06
131	ORF7b	DPA1*03:01/DPB1*04:02	IDFYLCFLAFLLFLV	98.31% (232/236)	Non-Toxin	Consensus (comb.lib./smm/nn)	0.16
132	ORF7b	DPA1*03:01/DPB1*04:02	LAFLLFLVLIMLIIF	99.58% (235/236)	Non-Toxin	Consensus (comb.lib./smm/nn)	0.08
133	ORF7b	DRB4*01:01	LAFLLFLVLIMLIIF	99.58% (235/236)	Non-Toxin	Consensus (comb.lib./smm/nn)	0.12
134	ORF7b	DPA1*01:03/DPB1*02:01	LIDFYLCFLAFLLFL	98.31% (232/236)	Non-Toxin	Consensus (comb.lib./smm/nn)	0.05
135	ORF7b	DPA1*01:03/DPB1*02:01	LSLIDFYLCFLAFLL	98.31% (232/236)	Non-Toxin	Consensus (comb.lib./smm/nn)	0.15
136	ORF7b	DQA1*01:01/DQB1*05:01	LVLIMLIIFWFSLEL	99.58% (235/236)	Non-Toxin	Consensus (comb.lib./smm/nn)	0.14
137	ORF7b	DPA1*01:03/DPB1*02:01	SLIDFYLCFLAFLLF	98.31% (232/236)	Non-Toxin	Consensus (comb.lib./smm/nn)	0.07
138	ORF8	DQA1*01:01/DQB1*05:01	LVVRCSFYEDFLEYH	99.58% (478/480)	Non-Toxin	Consensus (comb.lib./smm/nn)	0.45
139	ORF8	DRB3*01:01	QHQPYVVDDPCPIHF	99.17% (476/480)	Non-Toxin	Consensus (comb.lib./smm/nn)	0.08
140	ORF8	DRB3*01:01	TQHQPYVVDDPCPIH	99.17% (476/480)	Non-Toxin	Consensus (comb.lib./smm/nn)	0.08
141	ORF10	DPA1*01/DPB1*04:01	FAFPFTIYSLLLCRM	99.79% (478/479)	Non-Toxin	Consensus (comb.lib./smm)	0.56
142	ORF10	DPA1*02:01/DPB1*01:01	FAFPFTIYSLLLCRM	99.79% (478/479)	Non-Toxin	Consensus (comb.lib./smm/nn)	1.1
143	ORF10	DPA1*03:01/DPB1*04:02	FAFPFTIYSLLLCRM	99.79% (478/479)	Non-Toxin	Consensus (comb.lib./smm/nn)	1.1
144	ORF10	DPA1*01:03/DPB1*02:01	INVFAFPFTIYSLLL	99.37% (476/479)	Non-Toxin	Consensus (comb.lib./smm/nn)	0.29
145	ORF10	DPA1*01/DPB1*04:01	INVFAFPFTIYSLLL	99.37% (476/479)	Non-Toxin	Consensus (comb.lib./smm)	0.46
146	ORF10	DPA1*02:01/DPB1*01:01	INVFAFPFTIYSLLL	99.37% (476/479)	Non-Toxin	Consensus (comb.lib./smm/nn)	0.72
147	ORF10	DPA1*01:03/DPB1*02:01	NVFAFPFTIYSLLLC	99.58% (477/479)	Non-Toxin	Consensus (comb.lib./smm/nn)	0.4
148	ORF10	DPA1*01/DPB1*04:01	NVFAFPFTIYSLLLC	99.58% (477/479)	Non-Toxin	Consensus (comb.lib./smm)	0.56
149	ORF10	DPA1*02:01/DPB1*01:01	NVFAFPFTIYSLLLC	99.58% (477/479)	Non-Toxin	Consensus (comb.lib./smm/nn)	0.69
150	ORF10	DPA1*01/DPB1*04:01	VFAFPFTIYSLLLCR	99.58% (477/479)	Non-Toxin	Consensus (comb.lib./smm)	0.56
151	ORF10	DPA1*02:01/DPB1*01:01	VFAFPFTIYSLLLCR	99.58% (477/479)	Non-Toxin	Consensus (comb.lib./smm/nn)	0.66

152	ORF10	DPA1*01:03/DPB1*02:01	VFAFPFTIYSLLLCR	99.58% (477/479)	Non-Toxin	Consensus (comb.lib./smm/nn)	0.71
153	ORF10	DPA1*03:01/DPB1*04:02	VFAFPFTIYSLLLCR	99.58% (477/479)	Non-Toxin	Consensus (comb.lib./smm/nn)	1.1
154	S protein	DRB3*01:01	ADSFVIRGDEVRQIA	99.79% (471/472)	Non-Toxin	Consensus (comb.lib./smm/nn)	0.49
155	S protein	DRB1*07:01	AIPTNFTISVTTEIL	100.00% (472/472)	Non-Toxin	Consensus (comb.lib./smm/nn)	0.4
156	S protein	DRB3*01:01	DSFVIRGDEVRQIAP	99.79% (471/472)	Non-Toxin	Consensus (comb.lib./smm/nn)	0.51
157	S protein	DRB3*02:02	EGVFVSNGTHWFVTQ	99.36% (469/472)	Non-Toxin	NetMHCIIpan	0.21
158	S protein	DRB5*01:01	FVFKNIDGYFKIYSK	98.09% (463/472)	Non-Toxin	Consensus (smm/nn/sturniolo)	0.17
159	S protein	DRB1*11:01	GNYNYLYRLFRKSNL	98.52% (465/472)	Non-Toxin	Consensus (smm/nn/sturniolo)	0.22
160	S protein	DRB1*07:01	IAIPTNFTISVTTEI	100.00% (472/472)	Non-Toxin	Consensus (comb.lib./smm/nn)	0.47
161	S protein	DRB5*01:01	INITRFQTLLALHRS	99.36% (469/472)	Non-Toxin	Consensus (smm/nn/sturniolo)	0.32
162	S protein	DRB5*01:01	ITRFQTLLALHRSYL	99.36% (469/472)	Non-Toxin	Consensus (smm/nn/sturniolo)	0.26
163	S protein	DPA1*02:01/DPB1*14:01	ITRFQTLLALHRSYL	99.36% (469/472)	Non-Toxin	NetMHCIIpan	0.43
164	S protein	DRB1*13:02	LIVNNATNVVIKVCE	99.36% (469/472)	Non-Toxin	Consensus (smm/nn/sturniolo)	0.03
165	S protein	DRB1*01:01	LSFELLHAPATVCGP	98.09% (463/472)	Non-Toxin	Consensus (comb.lib./smm/nn)	0.03
166	S protein	DRB5*01:01	NITRFQTLLALHRSY	99.36% (469/472)	Non-Toxin	Consensus (smm/nn/sturniolo)	0.32
167	S protein	DPA1*02:01/DPB1*14:01	NITRFQTLLALHRSY	99.36% (469/472)	Non-Toxin	NetMHCIIpan	0.45
168	S protein	DRB1*07:01	PTNFTISVTTEILPV	100.00% (472/472)	Non-Toxin	Consensus (comb.lib./smm/nn)	0.51
169	S protein	DPA1*02:01/DPB1*14:01	QLIRAAEIRASANLA	99.36% (469/472)	Non-Toxin	NetMHCIIpan	0.31
170	S protein	DPA1*02:01/DPB1*14:01	QQLIRAAEIRASANL	99.36% (469/472)	Non-Toxin	NetMHCIIpan	0.2
171	S protein	DRB5*01:01	REFVFKNIDGYFKIY	97.88% (462/472)	Non-Toxin	Consensus (smm/nn/sturniolo)	0.17
172	S protein	DRB3*02:02	REGVFVSNGTHWFVT	99.58% (470/472)	Non-Toxin	NetMHCIIpan	0.2
173	S protein	DRB1*01:01	SFELLHAPATVCGPK	98.09% (463/472)	Non-Toxin	Consensus (comb.lib./smm/nn)	0.09
174	S protein	DRB3*01:01	SFVIRGDEVRQIAPG	99.79% (471/472)	Non-Toxin	Consensus (comb.lib./smm/nn)	0.51
175	S protein	DRB1*13:02	SKTQSLLIVNNATNV	100.00% (472/472)	Non-Toxin	Consensus (smm/nn/sturniolo)	0.03
176	S protein	DRB1*01:01	VLSFELLHAPATVCG	98.09% (463/472)	Non-Toxin	Consensus (comb.lib./smm/nn)	0.03
177	S protein	DRB1*01:01	VVLSFELLHAPATVC	98.09% (463/472)	Non-Toxin	Consensus (comb.lib./smm/nn)	0.03
178	S protein	DRB1*01:01	VVVLSFELLHAPATV	98.09% (463/472)	Non-Toxin	Consensus (comb.lib./smm/nn)	0.09
179	S protein	DRB3*01:01	YADSFVIRGDEVRQI	99.79% (471/472)	Non-Toxin	Consensus (comb.lib./smm/nn)	0.49
180	S protein	DQA1*05:01/DQB1*03:01	YIWLGFIAGLIAIVM	99.58% (470/472)	Non-Toxin	Consensus (comb.lib./smm/nn)	0.51

Supplementary table S11. Shortlisted B Cell epitopes (BepiPred Linear B Cell Prediction). B cell linear epitopes with length of 4 to 20 amino acids, predicted by the "BepiPred Linear B Cell Prediction" IEDB tool, from eleven SARS-CoV-2 proteins, are listed here. *In-silico* analysis has shown all the selected epitopes to be non-toxic (Non-Toxin) as well as they show significant amino acid sequence conservancy.

SARS-CoV-2 B Cell epitopes (Bepipred Linear Epitope Prediction)					
S.No.	Proteins	Peptide	Conservancy	Toxicity	Length
1	E protein	SEET	99.59% (480/482)	Non-Toxin	4
2	E protein	YVYSRVKNLNSSRVP	99.38% (479/482)	Non-Toxin	15
3	M protein	PLLESE	99.16% (473/477)	Non-Toxin	6
4	M protein	KLGASQRVAGDS	98.74% (471/477)	Non-Toxin	12
5	M protein	NGTITVEELKKLLEQW	98.53% (470/477)	Non-Toxin	16
6	M protein	YRIGNYKLNTDHSSSSDNIA	98.53% (470/477)	Non-Toxin	20
7	N protein	DPNFKD	97.19% (484/498)	Non-Toxin	6
8	N protein	AGLPYGANK	99.40% (495/498)	Non-Toxin	9
9	N protein	NGPQNQRNAPRI	96.18% (479/498)	Non-Toxin	12
10	N protein	SKQLQQSMSSADS	97.59% (486/498)	Non-Toxin	13
11	ORF1ab	IQYG	99.78% (455/456)	Non-Toxin	4
12	ORF1ab	YLKH	100% (456/456)	Non-Toxin	4
13	ORF1ab	RKVP	100% (456/456)	Non-Toxin	4
14	ORF1ab	YQCG	100% (456/456)	Non-Toxin	4
15	ORF1ab	ELSR	100% (456/456)	Non-Toxin	4
16	ORF1ab	TFCA	99.78% (455/456)	Non-Toxin	4
17	ORF1ab	GKIV	100% (456/456)	Non-Toxin	4
18	ORF1ab	EGNC	100% (456/456)	Non-Toxin	4
19	ORF1ab	LPYP	100% (456/456)	Non-Toxin	4
20	ORF1ab	SLRC	100% (456/456)	Non-Toxin	4
21	ORF1ab	KVQI	100% (456/456)	Non-Toxin	4
22	ORF1ab	HDIG	100% (456/456)	Non-Toxin	4
23	ORF1ab	WNAD	98.90% (451/456)	Non-Toxin	4
24	ORF1ab	EIPVA	99.56% (454/456)	Non-Toxin	5
25	ORF1ab	KGEDI	99.78% (455/456)	Non-Toxin	5
26	ORF1ab	NLYDK	100% (456/456)	Non-Toxin	5
27	ORF1ab	KFNPP	98.68% (450/456)	Non-Toxin	5
28	ORF1ab	GQQQT	98.46% (449/456)	Non-Toxin	5
29	ORF1ab	KFADD	99.34% (453/456)	Non-Toxin	5
30	ORF1ab	AAVNS	100% (456/456)	Non-Toxin	5
31	ORF1ab	SKCEE	99.12% (452/456)	Non-Toxin	5
32	ORF1ab	IEYTD	100% (456/456)	Non-Toxin	5
33	ORF1ab	IQPIG	100% (456/456)	Non-Toxin	5
34	ORF1ab	NSGSD	100% (456/456)	Non-Toxin	5
35	ORF1ab	EFTPF	100% (456/456)	Non-Toxin	5

36	ORF1ab	LLAKD	100% (456/456)	Non-Toxin	5
37	ORF1ab	EEMLD	100% (456/456)	Non-Toxin	5
38	ORF1ab	RSEDK	100% (456/456)	Non-Toxin	5
39	ORF1ab	TGHML	100% (456/456)	Non-Toxin	5
40	ORF1ab	AHSCN	99.78% (455/456)	Non-Toxin	5
41	ORF1ab	QTGSS	99.56% (454/456)	Non-Toxin	5
42	ORF1ab	LPVLQV	98.90% (451/456)	Non-Toxin	6
43	ORF1ab	FGDSVE	99.34% (453/456)	Non-Toxin	6
44	ORF1ab	ARTAPH	99.78% (455/456)	Non-Toxin	6
45	ORF1ab	AGKASC	100% (456/456)	Non-Toxin	6
46	ORF1ab	DFIDTK	99.78% (455/456)	Non-Toxin	6
47	ORF1ab	QYELKH	99.78% (455/456)	Non-Toxin	6
48	ORF1ab	FPDLNG	99.12% (452/456)	Non-Toxin	6
49	ORF1ab	SLDTYP	100% (456/456)	Non-Toxin	6
50	ORF1ab	SSFKWD	99.34% (453/456)	Non-Toxin	6
51	ORF1ab	KRNRAT	99.78% (455/456)	Non-Toxin	6
52	ORF1ab	KGFCKL	100% (456/456)	Non-Toxin	6
53	ORF1ab	AQVAKS	100% (456/456)	Non-Toxin	6
54	ORF1ab	VESSSK	99.34% (453/456)	Non-Toxin	6
55	ORF1ab	LYDKLQ	100% (456/456)	Non-Toxin	6
56	ORF1ab	ENKTTL	100% (456/456)	Non-Toxin	6
57	ORF1ab	DKGVAP	100% (456/456)	Non-Toxin	6
58	ORF1ab	ATNGPLK	100% (456/456)	Non-Toxin	7
59	ORF1ab	NKVENMT	99.78% (455/456)	Non-Toxin	7
60	ORF1ab	TRQVVNV	100% (456/456)	Non-Toxin	7
61	ORF1ab	EVGFVVP	100% (456/456)	Non-Toxin	7
62	ORF1ab	DASGKPV	100% (456/456)	Non-Toxin	7
63	ORF1ab	DYYRSLP	99.34% (453/456)	Non-Toxin	7
64	ORF1ab	GVSFSTF	100% (456/456)	Non-Toxin	7
65	ORF1ab	MAFPSGK	99.56% (454/456)	Non-Toxin	7
66	ORF1ab	SQGLLPP	99.56% (454/456)	Non-Toxin	7
67	ORF1ab	VQSKMSD	99.78% (455/456)	Non-Toxin	7
68	ORF1ab	AVDINKL	99.12% (452/456)	Non-Toxin	7
69	ORF1ab	DRDAAMQ	99.12% (452/456)	Non-Toxin	7
70	ORF1ab	FPKSDGT	100% (456/456)	Non-Toxin	7
71	ORF1ab	HPNPKGF	100% (456/456)	Non-Toxin	7
72	ORF1ab	GNKIADK	99.56% (454/456)	Non-Toxin	7
73	ORF1ab	YTPHTVL	99.78% (455/456)	Non-Toxin	7
74	ORF1ab	FEKGDYG	99.78% (455/456)	Non-Toxin	7
75	ORF1ab	VDSSQGS	100% (456/456)	Non-Toxin	7
76	ORF1ab	MYKGLPW	100% (456/456)	Non-Toxin	7
77	ORF1ab	IIGDELK	99.78% (455/456)	Non-Toxin	7
78	ORF1ab	QAWQPGV	99.78% (455/456)	Non-Toxin	7

79	ORF1ab	KPREQID	98.46% (449/456)	Non-Toxin	7
80	ORF1ab	VPGFNEKT	99.12% (452/456)	Non-Toxin	8
81	ORF1ab	RELNGGAY	100% (456/456)	Non-Toxin	8
82	ORF1ab	FTLKGGAP	99.78% (455/456)	Non-Toxin	8
83	ORF1ab	KTVGELGD	98.68% (450/456)	Non-Toxin	8
84	ORF1ab	NVPMEKLK	99.34% (453/456)	Non-Toxin	8
85	ORF1ab	GNATEVPA	100% (456/456)	Non-Toxin	8
86	ORF1ab	SGGQPITN	100% (456/456)	Non-Toxin	8
87	ORF1ab	EIIKSQDL	100% (456/456)	Non-Toxin	8
88	ORF1ab	YTVELGTEV	100% (456/456)	Non-Toxin	9
89	ORF1ab	IPTKKAGGT	100% (456/456)	Non-Toxin	9
90	ORF1ab	GLNGYTVEE	98.68% (450/456)	Non-Toxin	9
91	ORF1ab	YFYTSKTTV	100% (456/456)	Non-Toxin	9
92	ORF1ab	MSMTYGQQF	99.78% (455/456)	Non-Toxin	9
93	ORF1ab	AKNVSLDNV	99.78% (455/456)	Non-Toxin	9
94	ORF1ab	GGVTRDIAS	100% (456/456)	Non-Toxin	9
95	ORF1ab	VNLHSSRLS	100% (456/456)	Non-Toxin	9
96	ORF1ab	RNRDVDTDF	100% (456/456)	Non-Toxin	9
97	ORF1ab	LTKHPNQEY	100% (456/456)	Non-Toxin	9
98	ORF1ab	TEETFKLSY	98.90% (451/456)	Non-Toxin	9
99	ORF1ab	IPGIPKDMT	100% (456/456)	Non-Toxin	9
100	ORF1ab	FHTPAFDKS	98.24% (448/456)	Non-Toxin	9
101	ORF1ab	KGHFDGQQG	100% (456/456)	Non-Toxin	9
102	ORF1ab	GVVQQLPET	99.78% (455/456)	Non-Toxin	9
103	ORF1ab	IERYKLEGY	99.34% (453/456)	Non-Toxin	9
104	ORF1ab	VEKGVLPQLE	99.78% (455/456)	Non-Toxin	10
105	ORF1ab	SEKSYELQTP	99.56% (454/456)	Non-Toxin	10
106	ORF1ab	CHNSEVGPEH	99.12% (452/456)	Non-Toxin	10
107	ORF1ab	CVKSREETGL	99.56% (454/456)	Non-Toxin	10
108	ORF1ab	SYKDWSYSGQ	99.78% (455/456)	Non-Toxin	10
109	ORF1ab	ALLTKSSEYK	99.78% (455/456)	Non-Toxin	10
110	ORF1ab	QLTGYKKPAS	99.56% (454/456)	Non-Toxin	10
111	ORF1ab	ILKPANNSLK	99.56% (454/456)	Non-Toxin	10
112	ORF1ab	YNYEPLTQDH	98.24% (448/456)	Non-Toxin	10
113	ORF1ab	EQAVANGDSE	99.78% (455/456)	Non-Toxin	10
114	ORF1ab	PEANMDQESF	99.78% (455/456)	Non-Toxin	10
115	ORF1ab	PCGTGTSTDV	97.58% (445/456)	Non-Toxin	10
116	ORF1ab	FQEKDEDDNL	98.90% (451/456)	Non-Toxin	10
117	ORF1ab	TFSNYQHEET	100% (456/456)	Non-Toxin	10
118	ORF1ab	FFKFRIDGDM	99.56% (454/456)	Non-Toxin	10
119	ORF1ab	LKYAISAKNR	99.78% (455/456)	Non-Toxin	10
120	ORF1ab	YASQGLVASI	99.56% (454/456)	Non-Toxin	10
121	ORF1ab	WTETDLTKGP	99.78% (455/456)	Non-Toxin	10

122	ORF1ab	QVNGYPNMFI	100% (456/456)	Non-Toxin	10
123	ORF1ab	YKRDAPAHIS	100% (456/456)	Non-Toxin	10
124	ORF1ab	RVDGQVDLFR	98.90% (451/456)	Non-Toxin	10
125	ORF1ab	HTKKWKYPQVN	99.12% (452/456)	Non-Toxin	11
126	ORF1ab	KRPINPTDQSS	99.34% (453/456)	Non-Toxin	11
127	ORF1ab	LFSTVFPPTSF	72.58% (331/456)	Non-Toxin	11
128	ORF1ab	VGKPRPPLNRN	99.34% (453/456)	Non-Toxin	11
129	ORF1ab	RVLSNLNLPGC	98.90% (451/456)	Non-Toxin	11
130	ORF1ab	DIAKKPTETIC	99.78% (455/456)	Non-Toxin	11
131	ORF1ab	YHNESGLKTILR	99.78% (455/456)	Non-Toxin	12
132	ORF1ab	EGETLPTEVLTE	99.56% (454/456)	Non-Toxin	12
133	ORF1ab	KHYTPSFKKGAK	99.56% (454/456)	Non-Toxin	12
134	ORF1ab	RQGFVDSDVETK	99.78% (455/456)	Non-Toxin	12
135	ORF1ab	SDVLLPLTQYNR	99.78% (455/456)	Non-Toxin	12
136	ORF1ab	KLQNNELSPVAL	99.56% (454/456)	Non-Toxin	12
137	ORF1ab	MHAASGNLLLDK	99.78% (455/456)	Non-Toxin	12
138	ORF1ab	KPGGTSSGDATT	100% (456/456)	Non-Toxin	12
139	ORF1ab	TNDNTSRYWEPE	100% (456/456)	Non-Toxin	12
140	ORF1ab	PYVCNAPGCDVT	100% (456/456)	Non-Toxin	12
141	ORF1ab	GCHATREAVGTN	98.24% (448/456)	Non-Toxin	12
142	ORF1ab	SVKGLQPSVGPK	99.78% (455/456)	Non-Toxin	12
143	ORF1ab	LNDFVSDADSTL	99.78% (455/456)	Non-Toxin	12
144	ORF1ab	YILPSIISNEKQE	100% (456/456)	Non-Toxin	13
145	ORF1ab	GLNLEEAARYMRS	100% (456/456)	Non-Toxin	13
146	ORF1ab	FDNLKTLLSLREV	99.78% (455/456)	Non-Toxin	13
147	ORF1ab	EQFKKGVQIPCTC	99.56% (454/456)	Non-Toxin	13
148	ORF1ab	EDMLNPNYEDLLI	99.56% (454/456)	Non-Toxin	13
149	ORF1ab	RNVATLQAENVTG	100% (456/456)	Non-Toxin	13
150	ORF1ab	PKTKNVTKENDSK	98.90% (451/456)	Non-Toxin	13
151	ORF1ab	YNKYKYFSGAMDTT	99.56% (454/456)	Non-Toxin	14
152	ORF1ab	EEAIRHVRAWIGFD	100% (456/456)	Non-Toxin	14
153	ORF1ab	CGETSWQTGDFVKAT	99.34% (453/456)	Non-Toxin	15
154	ORF1ab	LQPLEQPTSEAVEAP	99.56% (454/456)	Non-Toxin	15
155	ORF1ab	VSELLTPLGIDLDEW	99.78% (455/456)	Non-Toxin	15
156	ORF1ab	SKHTDFSSEIIGYKA	98.90% (451/456)	Non-Toxin	15
157	ORF1ab	PAPRTLLTKGTLEPE	99.34% (453/456)	Non-Toxin	15
158	ORF1ab	KRNIKPVPEVKILNN	98.90% (451/456)	Non-Toxin	15
159	ORF1ab	RFKESPFELEDFIPM	99.34% (453/456)	Non-Toxin	15
160	ORF1ab	GTENLTKEGATTCGYLP	99.12% (452/456)	Non-Toxin	17
161	ORF1ab	DFDTWFSQRGGSYTNDK	99.78% (455/456)	Non-Toxin	17
162	ORF1ab	TANPKTPKYKFVRIQPG	99.12% (452/456)	Non-Toxin	17
163	ORF1ab	DDYFNKKDWYDFVENPD	98.90% (451/456)	Non-Toxin	17
164	ORF1ab	LQKEKVNINIVGDFKLNE	100% (456/456)	Non-Toxin	18

165	ORF1ab	WDTIANYAKPFLNKVVST	98.68% (450/456)	Non-Toxin	18
166	ORF1ab	AVTAYNGYLTSSSKTPEEH	100% (456/456)	Non-Toxin	19
167	ORF1ab	GCSCDQLREPMLQSADAQS	98.90% (451/456)	Non-Toxin	19
168	ORF1ab	PNNTDFSRVSAKPPPGDQF	99.78% (455/456)	Non-Toxin	19
169	ORF1ab	RMLLEKCDLQNYGDSATLP	100% (456/456)	Non-Toxin	19
170	ORF1ab	IVSTIQRKYKGIKIQEGVVD	99.12% (452/456)	Non-Toxin	20
171	ORF1ab	GTTQTACTDDNALAYYNTTK	99.78% (455/456)	Non-Toxin	20
172	ORF1ab	IQLSSYSLFDMSKFPLKLRG	98.46% (449/456)	Non-Toxin	20
173	ORF3a	SKNPLL	96.25% (463/481)	Non-Toxin	6
174	ORF3a	PYNSVT	97.92% (471/481)	Non-Toxin	6
175	ORF3a	STQLSTDTGV	99.58%(479/481)	Non-Toxin	10
176	ORF3a	KIITLKKRWQL	99.37% (478/481)	Non-Toxin	11
177	ORF3a	QGEIKDATPSDF	99.37% (478/481)	Non-Toxin	12
178	ORF6	LTENKYSQLDEEQP	99.17% (477/481)	Non-Toxin	14
179	ORF7a	LYHYQECVR	99.79% (479/480)	Non-Toxin	9
180	ORF7b	ELQDHNE	100.00% (236/236)	Non-Toxin	7
181	ORF8	QEPKL	100.00% (480/480)	Non-Toxin	5
182	ORF8	EDFLEY	99.79% (479/480)	Non-Toxin	6
183	ORF8	RVGARKSAP	99.17% (476/480)	Non-Toxin	9
184	ORF8	DEAGSKSPIQYIDIGN	98.96% (475/480)	Non-Toxin	16
185	S protein	LDPL	99.58% (470/472)	Non-Toxin	4
186	S protein	QTLE	100.00% (472/472)	Non-Toxin	4
187	S protein	LGKY	100.00% (472/472)	Non-Toxin	4
188	S protein	TNTSN	100.00% (472/472)	Non-Toxin	5
189	S protein	EAEVQ	99.15% (468/472)	Non-Toxin	5
190	S protein	EQDKNTQ	99.15% (468/472)	Non-Toxin	7
191	S protein	SNKKFLPF	100.00% (472/472)	Non-Toxin	8
192	S protein	PDPSKPSK	98.31% (464/472)	Non-Toxin	8
193	S protein	GQSKRVDFC	100.00% (472/472)	Non-Toxin	9
194	S protein	TPGDSSSGWTA	99.58% (470/472)	Non-Toxin	11
195	S protein	RVYSTGSNVFQ	99.79% (471/472)	Non-Toxin	11
196	S protein	VNNSYECDIPI	100.00% (472/472)	Non-Toxin	11
197	S protein	RNFYEPQIITTD	99.36% (469/472)	Non-Toxin	12
198	S protein	MDLEGKQGNFKNL	98.52% (465/472)	Non-Toxin	13
199	S protein	KQIYKTPPIKDFGGF	98.09% (463/472)	Non-Toxin	15
200	S protein	LADAGFIKQYGDCLG	99.36% (469/472)	Non-Toxin	15
201	S protein	KHTPINLVRDLPQGFS	99.58% (470/472)	Non-Toxin	16
202	S protein	YTMSLGAENSVAYSNN	100.00% (472/472)	Non-Toxin	16
203	S protein	DPFLGVYYHKNNKSWME	98.09% (463/472)	Non-Toxin	17
204	S protein	NCTEVPVAIHADQLTPT	100.00% (472/472)	Non-Toxin	17
205	S protein	KSFTVEKGIYQTSNFRVQP	99.36% (469/472)	Non-Toxin	19
206	S protein	ASYQTQTNSPRRARSVASQ	100.00% (472/472)	Non-Toxin	19

Supplementary table S12. World population coverage by the shortlisted SARS-CoV-2 CTL and HTL epitopes combined. With a standard deviation of 23.74 on an average 96.10 % of the world population could be covered by the joint administration of selected CTL and HTL epitopes (given in Table 1 and 2) as vaccine candidates.

Population/area	Class I and II combined		
	% coverage[a]	average_hit[b]	pc90[c]
Algeria	27.85%	1.51	0.69
American Samoa	70.35%	1.93	0.34
Argentina	96.78%	3.56	1.35
Australia	79.62%	2.73	0.49
Austria	99.28%	7.17	2.74
Belgium	97.21%	4.63	1.71
Bolivia	35.81%	2.14	0.77
Brazil	92.28%	3.69	1.15
Bulgaria	95.49%	4.48	1.64
Burkina Faso	61.26%	1.3	0.26
Cameroon	88.90%	2.71	0.9
Cape Verde	98.02%	5.71	2.07
Central Africa	87.66%	3.35	0.81
Central African Republic	36.40%	1.25	0.16
Chile	90.76%	4.36	1.06
China	90.43%	4.74	1.05
Colombia	24.90%	1.18	0.27
Congo	29.65%	1.58	0.64
Cook Islands	22.52%	1.33	0.65
Croatia	97.46%	5.28	1.97
Cuba	97.36%	6.08	2.03
Czech Republic	98.68%	5.99	2.31
Denmark	59.30%	3.61	1.23
East Africa	90.29%	3.46	1.02
East Asia	93.23%	5.49	1.29
England	99.45%	7.37	3.14
Equatorial Guinea	15.47%	0.81	0.58
Ethiopia	34.14%	1.72	0.71
Europe	98.35%	6.12	2.32
Finland	99.58%	5.27	2.46
France	98.20%	6.39	2.31
Georgia	93.92%	4.08	1.31
Germany	99.34%	7.17	2.92
Greece	26.34%	1.51	0.68

Guinea-Bissau	95.03%	4.49	1.37
Hong Kong	84.82%	2.7	0.66
India	86.82%	3.86	0.76
Indonesia	78.44%	2.93	0.46
Iran	94.14%	4.47	1.45
Ireland Northern	99.55%	7.7	3.3
Ireland South	99.28%	7.31	3.16
Israel	84.33%	3.49	0.64
Italy	96.70%	4.11	1.74
Ivory Coast	65.09%	1.16	0.29
Japan	92.78%	5.3	1.23
Jordan	85.10%	3.54	0.67
Kenya	86.65%	2.27	0.75
Korea; South	95.79%	6.18	1.72
Macedonia	43.69%	2.18	0.18
Malaysia	80.94%	3.79	0.52
Mali	95.75%	2.67	1.3
Martinique	43.47%	1.8	0.18
Mexico	93.68%	3.6	1.35
Mongolia	87.03%	4.25	0.77
Morocco	97.32%	5.55	2.01
Netherlands	40.49%	2.35	0.84
New Caledonia	61.85%	2.73	0.26
New Zealand	24.11%	1.39	0.66
Niue	20.26%	1.2	0.63
North Africa	94.14%	4.19	1.3
North America	97.36%	6	2.09
Northeast Asia	90.30%	4.71	1.04
Norway	52.39%	3.08	1.05
Oceania	75.64%	3.29	0.41
Oman	94.27%	3.2	1.35
Pakistan	85.00%	2.54	0.67
Papua New Guinea	71.23%	3.29	0.35
Peru	95.12%	3.38	1.52
Philippines	67.23%	1.92	0.61
Poland	98.46%	6.18	2.36
Portugal	96.27%	5.25	1.75
Romania	95.90%	3.54	1.56
Russia	94.88%	5.26	1.49
Rwanda	45.12%	1.85	0.18
Samoa	43.64%	2.65	0.89
Sao Tome and Principe	96.32%	4.15	1.54
Saudi Arabia	96.53%	4.87	1.65
Scotland	73.06%	4.19	0.37

Senegal	94.11%	3.54	1.24
Serbia	62.91%	1.07	0.27
Singapore	86.92%	4.37	0.76
Slovenia	43.37%	2.58	0.88
South Africa	88.81%	2.6	0.89
South America	81.68%	2.95	0.55
South Asia	90.37%	4.25	1.03
Southeast Asia	82.33%	3.79	0.57
Southwest Asia	86.38%	3.13	0.73
Spain	87.57%	3.8	0.8
Sri Lanka	52.39%	1.59	0.42
Sudan	87.98%	2.69	0.83
Sweden	99.29%	7.32	2.59
Taiwan	84.93%	4.28	0.66
Thailand	91.26%	4.81	1.17
Tonga	33.43%	1.98	0.69
Tunisia	94.64%	4.17	1.38
Turkey	61.05%	2.54	0.51
Uganda	92.00%	2.99	1.13
United States	97.45%	6.04	2.11
Venezuela	69.56%	1.16	0.33
Vietnam	86.19%	4.11	0.72
West Africa	94.34%	4.44	1.32
West Indies	96.71%	5.04	1.74
Zambia	97.50%	3.1	1.67
Zimbabwe	93.60%	3.4	1.21
World	96.10%	5.55	1.8
Average	78.62	3.73	1.16
Standard deviation	23.74	1.67	0.73

[a] projected population coverage
[b] average number of epitope hits / HLA combinations recognized by the population
[c] minimum number of epitope hits / HLA combinations recognized by 90% of the population

Supplementary Fig. S1. Workflow chart.

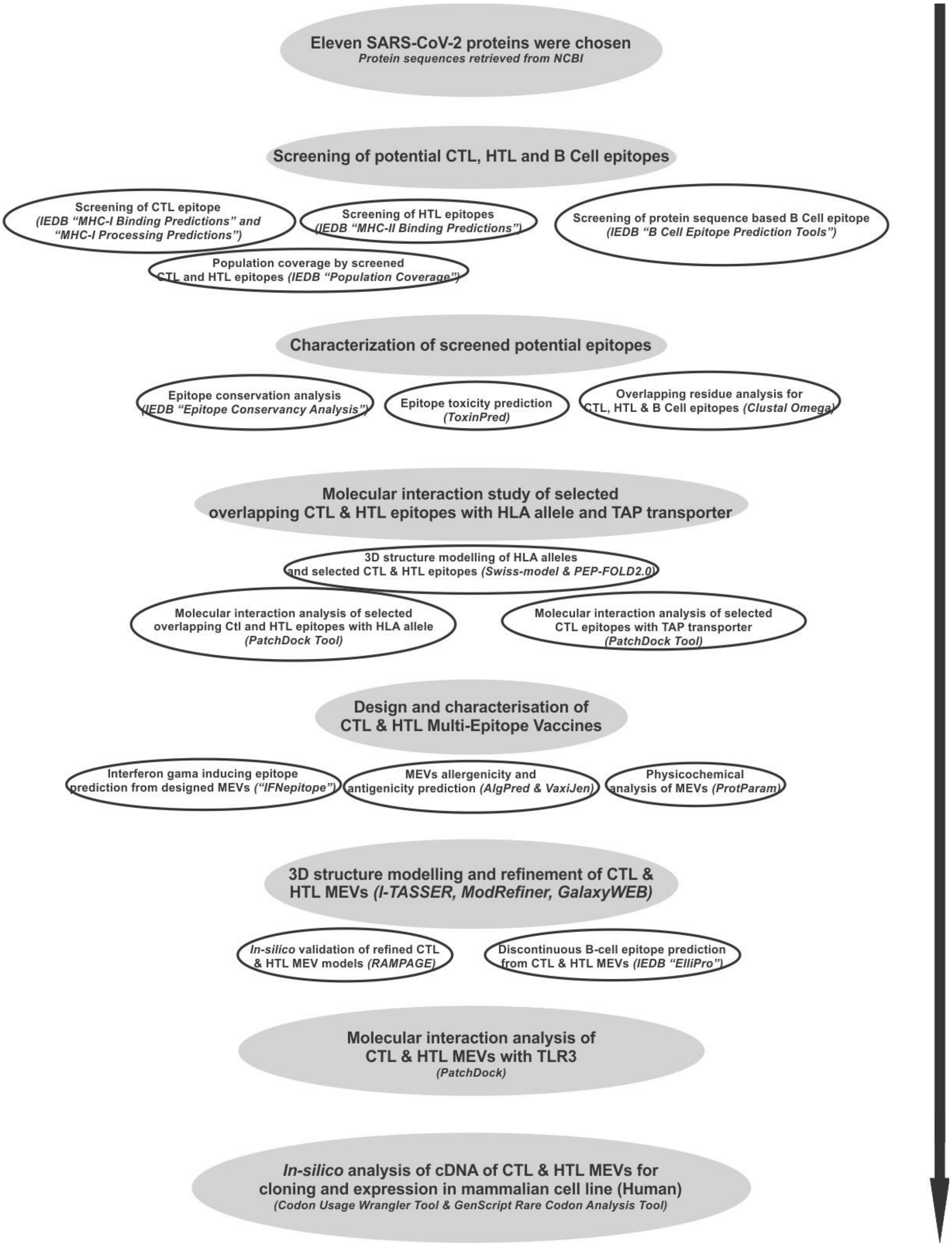

Supplementary Fig. S2. Design of SARS-CoV-2 Multi-Epitope Vaccine (MEVs). (A) CTL and (B) HTL epitopes were linked by the short peptide linker 'GGGGS'. Truncated (residues 10-153) Onchocerca volvulus activation-associated secreted protein-1 (Ov-ASP-1) has been utilized at the N terminal of both the MEVs. The short peptide EAAAK was used to link Ov-ASP-1 at N terminal. Epitopes from different proteins were colored in different colors. C terminal 6xHis is designed as His tag.

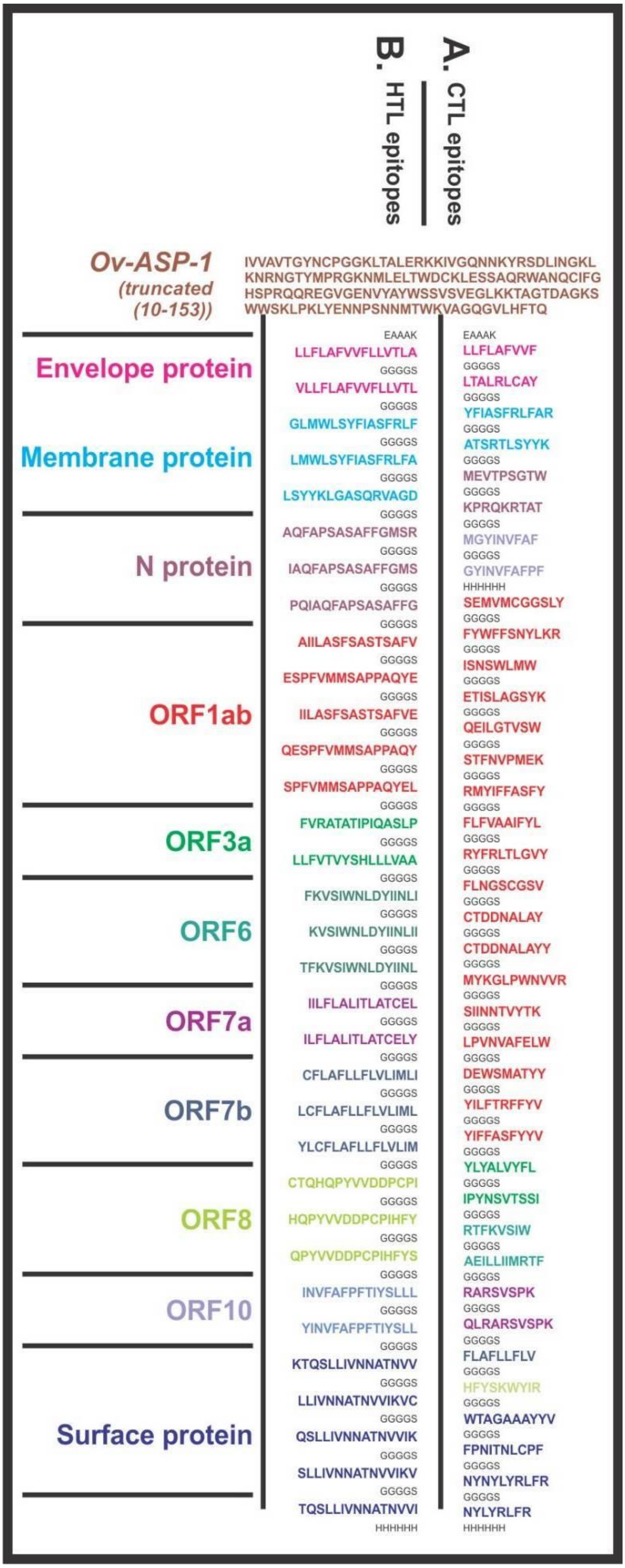

A. CTL epitopes
B. HTL epitopes
Ov-ASP-1
(truncated (10-153))
IVVAVTGYNCPGGKLTALERKKIVGQNNKYRSDLINGKL
KNRNGTYMPRGKNMLELTWDCKLESSAQRWANQCIFG
HSPRQQREGVGENVYAYWSSVSVEGLKKTAGTDAGKS
WWSKLPKLYENNPSNNMTWKVAGQGVLHFTQ
Envelope protein
Membrane protein
N protein
ORF1ab
ORF3a
ORF6
ORF7a
ORF7b
ORF8
ORF10
Surface protein
EAAAK
LLFLAFVVFLLVTLA
GGGGS
VLLFLAFVVFLLVTL
GGGGS
GLMWLSYFIASFRLF
GGGGS
LMWLSYFIASFRLFA
GGGGS
LSYYKLGASQRVAGD
GGGGS
AQFAPSASAFFGMSR
GGGGS
IAQFAPSASAFFGMS
GGGGS
PQIAQFAPSASAFFG
GGGGS
AIILASFSASTSAFV
GGGGS
ESPFVMMSAPPAQYE
GGGGS
IILASFSASTSAFVE
GGGGS
QESPFVMMSAPPAQY
GGGGS
SPFVMMSAPPAQYEL
GGGGS
FVRATATIPIQASLP
GGGGS
LLFVTVYSHLLLVAA
GGGGS
FKVSIWNLDYIINLI
GGGGS
KVSIWNLDYIINLII
GGGGS
TFKVSIWNLDYIINL
GGGGS
IILFLALITLATCEL
GGGGS
ILFLALITLATCELY
GGGGS
CFLAFLLFLVLIMLI
GGGGS
LCFLAFLLFLVLIML
GGGGS
YLCFLAFLLFLVLIM
GGGGS
CTQHQPYVVDDPCPI
GGGGS
HQPYVVDDPCPIHFY
GGGGS
QPYVVDDPCPIHFYS
GGGGS
INVFAFPFTIYSLLL
GGGGS
YINVFAFPFTIYSLL
GGGGS
KTQSLLIVNNATNVV
GGGGS
LLIVNNATNVVIKVC
GGGGS
QSLLIVNNATNVVIK
GGGGS
SLLIVNNATNVVIKV
GGGGS
TQSLLIVNNATNVVI
HHHHHH
EAAAK
LLFLAFVVF
GGGGS
LTALRLCAY
GGGGS
YFIASFRLFAR
GGGGS
ATSRTLSYYK
GGGGS
MEVTPSGTW
GGGGS
KPRQKRTAT
GGGGS
MGYINVFAF
GGGGS
GYINVFAFPF
HHHHHH
SEMVMCGGSLY
GGGGS
FYWFFSNYLKR
GGGGS
ISNSWLMW
GGGGS
ETISLAGSYK
GGGGS
QEILGTVSW
GGGGS
STFNVPMEK
GGGGS
RMYIFFASFY
GGGGS
FLFVAAIFYL
GGGGS
RYFRLTLGVY
GGGGS
FLNGSCGSV
GGGGS
CTDDNALAY
GGGGS
CTDDNALAYY
GGGGS
MYKGLPWNVVR
GGGGS
SIINNTVYTK
GGGGS
LPVNVAFELW
GGGGS
DEWSMATYY
GGGGS
YILFTRFFYV
GGGGS
YIFFASFYYV
GGGGS
YLYALVYFL
GGGGS
IPYNSVTSSI
GGGGS
RTFKVSIW
GGGGS
AEILLIIMRTF
GGGGS
RARSVSPK
GGGGS
QLRARSVSPK
GGGGS
FLAFLLFLV
GGGGS
HFYSKWYIR
GGGGS
WTAGAAAYYV
GGGGS
FPNITNLCPF
GGGGS
NYNYLYRLFR
GGGGS
NYLYRLFR
HHHHHH

Supplementary Fig. S3. Overlapping SARS-CoV-2 CTL, HTL and B cell epitopes. Multiple sequence alignment performed by Clustal Omega at EBI to identify the consensus overlapping regions of CTL (red), HTL (blue) and B cell epitopes (green) amongst shortlisted epitopes. Epitopes with overlapping regions amongst all the three types of epitopes (CTL, HTL and B Cell epitopes) were chosen for further studies (encircled).

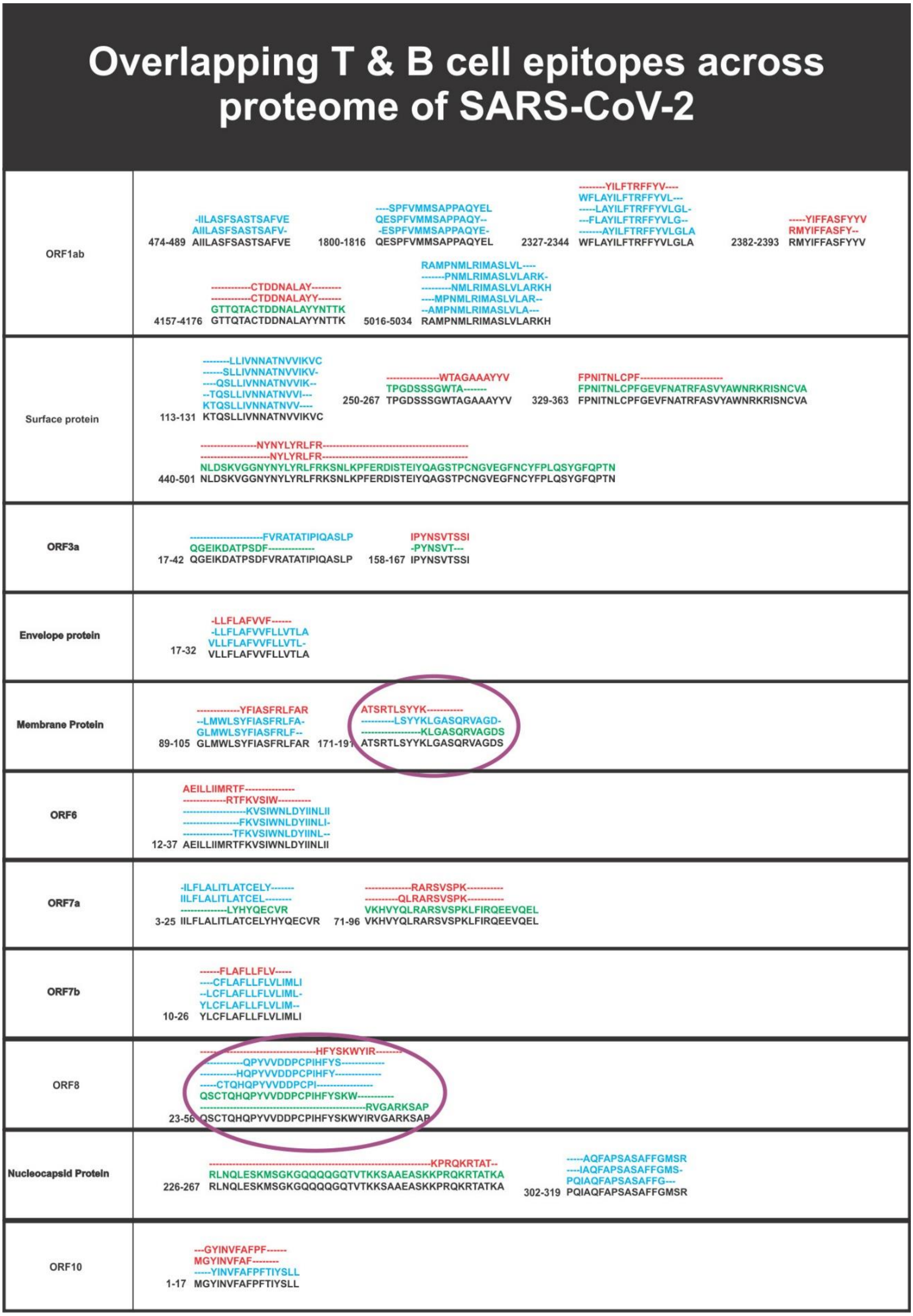
Overlapping T & B cell epitopes across proteome of SARS-CoV-2
ORF1ab
-IILASFSASTSAFVE
AIILASFSASTSAFV-
474-489 AIILASFSASTSAFVE
----SPFVMMSAPPAQYEL
QESPFVMMSAPPAQY--
-ESPFVMMSAPPAQYE-
1800-1816 QESPFVMMSAPPAQYEL
--------YILFTRFFYV----
WFLAYILFTRFFYVL---
-----LAYILFTRFFYVLGL-
---FLAYILFTRFFYVLG--
-------AYILFTRFFYVLGLA
2327-2344 WFLAYILFTRFFYVLGLA
-----YIFFASFYYV
RMYIFFASFY--
2382-2393 RMYIFFASFYYV
------------CTDDNALAY---------
-----------CTDDNALAYY-------
GTTQTACTDDNALAYYNTTK
4157-4176 GTTQTACTDDNALAYYNTTK
RAMPNMLRIMASLVL----
-------PNMLRIMASLVLARK-
---------NMLRIMASLVLARKH
----MPNMLRIMASLVLAR--
--AMPNMLRIMASLVLA---
5016-5034 RAMPNMLRIMASLVLARKH
Surface protein
--------LLIVNNATNVVIKVC
------SLLIVNNATNVVIKV-
----QSLLIVNNATNVVIK--
--TQSLLIVNNATNVVI---
KTQSLLIVNNATNVV----
113-131 KTQSLLIVNNATNVVIKVC
---------------WTAGAAAYYV
TPGDSSSGWTA-------
250-267 TPGDSSSGWTAGAAAYYV
FPNITNLCPF------------------------
FPNITNLCPFGEVFNATRFASVYAWNRKRISNCVA
329-363 FPNITNLCPFGEVFNATRFASVYAWNRKRISNCVA
-----------------NYNYLYRLFR--
--------------------NYLYRLFR--
NLDSKVGGNYNYLYRLFRKSNLKPFERDISTEIYQAGSTPCNGVEGFNCYFPLQSYGFQPTN
440-501 NLDSKVGGNYNYLYRLFRKSNLKPFERDISTEIYQAGSTPCNGVEGFNCYFPLQSYGFQPTN
ORF3a
----------------------FVRATATIPIQASLP
QGEIKDATPSDF--------------
17-42 QGEIKDATPSDFVRATATIPIQASLP
IPYNSVTSSI
-PYNSVT---
158-167 IPYNSVTSSI
Envelope protein
-LLFLAFVVF------
-LLFLAFVVFLLVTLA
VLLFLAFVVFLLVTL-
17-32 VLLFLAFVVFLLVTLA
Membrane Protein
-------------YFIASFRLFAR
--LMWLSYFIASFRLFA-
GLMWLSYFIASFRLF--
89-105 GLMWLSYFIASFRLFAR
ATSRTLSYYK-----------
----------LSYYKLGASQRVAGD-
------------------KLGASQRVAGDS
171-191 ATSRTLSYYKLGASQRVAGDS
ORF6
AEILLIIMRTF---------------
-------------RTFKVSIW----------
-------------------KVSIWNLDYIINLII
-----------------FKVSIWNLDYIINLI-
---------------TFKVSIWNLDYIINL--
12-37 AEILLIIMRTFKVSIWNLDYIINLII
ORF7a
-ILFLALITLATCELY-------
IILFLALITLATCEL--------
--------------LYHYQECVR
3-25 IILFLALITLATCELYHYQECVR
--------------RARSVSPK-----------
----------QLRARSVSPK-----------
VKHVYQLRARSVSPKLFIRQEEVQEL
71-96 VKHVYQLRARSVSPKLFIRQEEVQEL
ORF7b
------FLAFLLFLV-----
----CFLAFLLFLVLIMLI
--LCFLAFLLFLVLIML-
YLCFLAFLLFLVLIM--
10-26 YLCFLAFLLFLVLIMLI
ORF8
---------------------------------HFYSKWYIR--------
-----------QPYVVDDPCPIHFYS-------------
-----------HQPYVVDDPCPIHFY-------------
-----CTQHQPYVVDDPCPI-----------------
QSCTQHQPYVVDDPCPIHFYSKW-----------
--RVGARKSAP
23-56 QSCTQHQPYVVDDPCPIHFYSKWYIRVGARKSAP
Nucleocapsid Protein
---KPRQKRTAT--
RLNQLESKMSGKGQQQQGQTVTKKSAAEASKKPRQKRTATKA
226-267 RLNQLESKMSGKGQQQQGQTVTKKSAAEASKKPRQKRTATKA
-----AQFAPSASAFFGMSR
----IAQFAPSASAFFGMS-
PQIAQFAPSASAFFG---
302-319 PQIAQFAPSASAFFGMSR
ORF10
---GYINVFAFPF------
MGYINVFAF--------
-----YINVFAFPFTIYSLL
1-17 MGYINVFAFPFTIYSLL

Supplementary Fig. S4. B-Factor of CTL and HTL epitope in complex with HLA class I and II allele. CTL and HTL Epitopes are shown in sticks and HLA Class I and II alleles are shown in ribbon. The HLA alleles are shown in gray. The regions of the epitope in the complex are shown in a rainbow (VIBGYOR), the regions in blue being very stable and the region towards red being relatively unstable. In the complexes shown above, most of the regions of epitopes are in blue indicating the complexes to be highly stable.

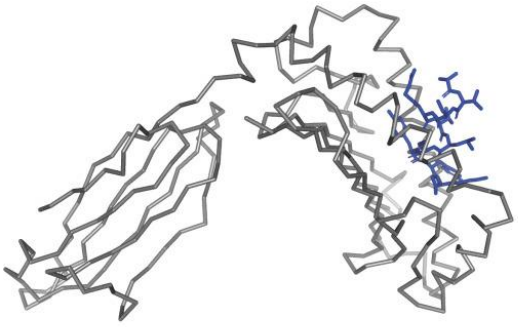

1. ATSRTLSYYK-HLA-A*11:01

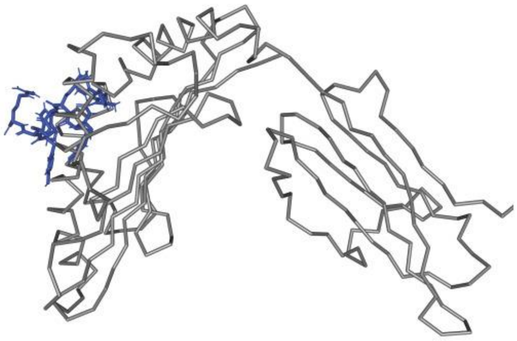

2. HFYSKWYIR-HLA-A*31:01

CTL epitopes - HLA Class I allele complexes

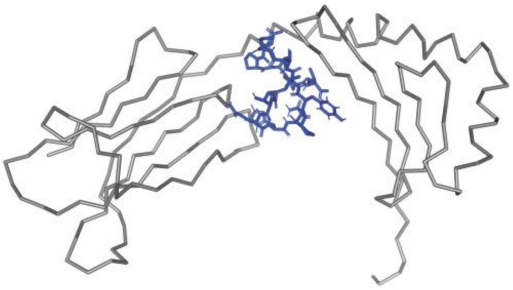

3. CTQHQPYVVDDPCPI-HLA-DRB3*01:01

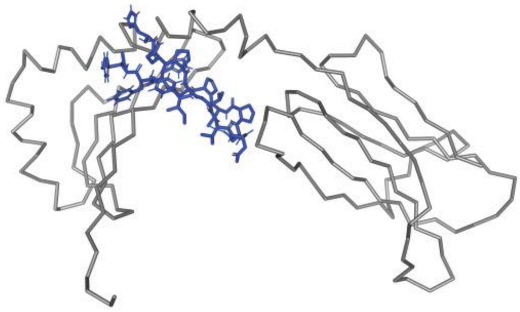

4. HQPYVVDDPCPIHFY-HLA-DRB3*01:01

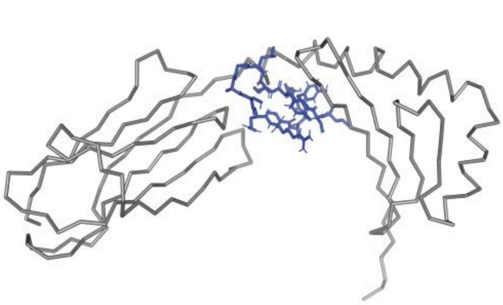

5. LSYYKLGASQRVAGD-HLA-DRB1*09:01

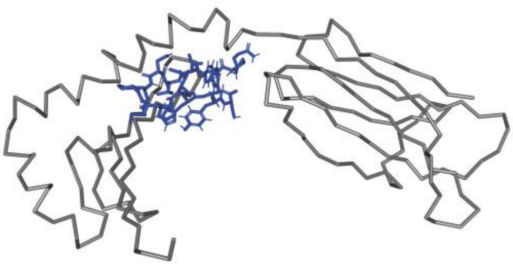

6. QPYVVDDPCPIHFYS-HLA-DRB3*01:01

HTL epitopes - HLA Class II allele complexes

Supplementary Fig. S5. RAMPAGE analysis of CTL and HTL MEVs. The RAMPAGE analysis of both the CTL and HTL MEVs has been done and shown here.

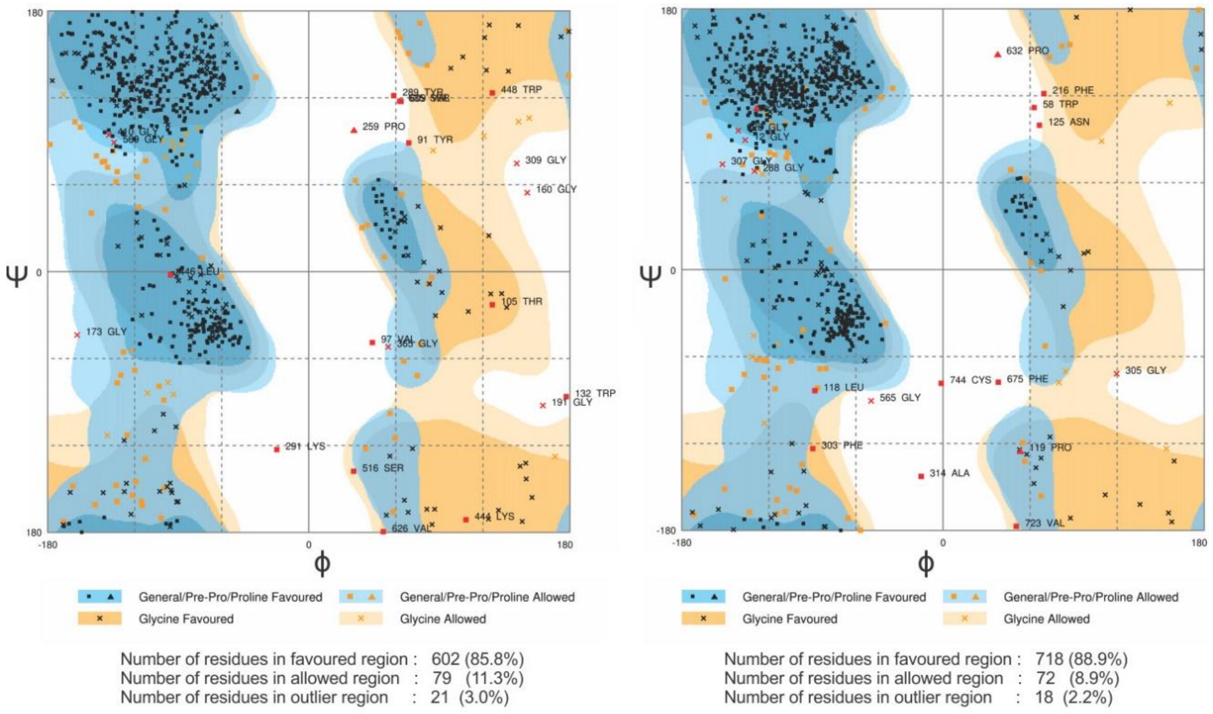

A. CTL Multi-epitope vaccine model **B. HTL Multi-epitope vaccine model**
